# From Triplex to Quadruplex: Enhancing CDC’s Respiratory qPCR Assay with RSV Detection on Panther Fusion^®^ Open Access™

**DOI:** 10.3390/microorganisms14010167

**Published:** 2026-01-12

**Authors:** Andy Caballero Méndez, Mayeline N. Sosa Ortiz, Roberto A. Reynoso de la Rosa, Miguel E. Abreu Bencosme, Karla V. Montero Lebrón

**Affiliations:** Molecular Biology Department, Referencia Laboratorio Clínico, Santo Domingo Oeste, Santo Domingo 11005, Dominican Republic

**Keywords:** RT-qPCR, multiplex assay, respiratory viruses, SARS-CoV-2, influenza A/B, respiratory syncytial virus, Panther Fusion^®^ Open Access™, laboratory-developed test (LDT), performance evaluation, molecular diagnostics

## Abstract

The overlapping circulation of influenza (Flu), severe acute respiratory syndrome coronavirus 2 (SARS-CoV-2; SC2), and respiratory syncytial virus (RSV) continues to challenge clinical laboratories, particularly in settings with limited automation and fragmented healthcare coverage. This study expanded the CDC Flu-SC2 assay by incorporating a laboratory-developed test (LDT) for RSV A/B detection into a fully automated quadruplex RT-qPCR (LDRA) on the Panther Fusion^®^ Open Access™ system. The design, based on more than 8000 RSV genomic sequences targeting the conserved M gene, achieved optimal amplification efficiencies (97–105%) and full multiplex compatibility. Analytical assessment established limits of detection between 9.6 and 37.8 copies per reaction, absence of cross-reactivity with 30 respiratory pathogens, and inclusivity for 32 viral variants. Commutability and diagnostic performance among the LDRA, CE IVD-marked Allplex™ SARS-CoV-2/FluA/FluB/RSV, and US IVD-marked Panther Fusion^®^ SARS-CoV-2/Flu A/B/RSV Assays were evaluated using 405 nasopharyngeal UTM-preserved swabs. The LDRA demonstrated excellent concordance (overall agreement ≥ 98%, *κ* > 0.95), strong diagnostic accuracy, and reliable detection of mixed infections. This quadruplex provides a fully automated, rapid, and accurate solution for the simultaneous detection of influenza A, influenza B, SARS-CoV-2, and RSV viruses, enhancing molecular diagnostic capacity and supporting equitable, timely clinical decision-making in middle-income healthcare systems such as that of the Dominican Republic.

## 1. Introduction

The coronavirus disease 2019 pandemic (COVID-19) has dramatically altered the epidemiology of other respiratory viruses. Between 2020 and 2021, the traditionally endemic influenza A (IAV), influenza B (IBV), and respiratory syncytial virus (RSV) were virtually absent due to public health measures aimed at containing the spread of severe acute respiratory syndrome coronavirus 2 (SARS-CoV-2; SC2) [1,2]. In the post-pandemic period, as previously anticipated [3], influenza, SC2, and RSV infections represent a global public health threat due to their high transmissibility, morbidity, recurrent seasonal waves, and economic burden [4,5].

The overlapping symptoms of respiratory infections pose an additional challenge to healthcare systems by increasing the risk of complications and hindering therapeutic strategies [6,7]. These findings underscore the need to strengthen surveillance, diagnosis, and prevention systems, particularly for vulnerable populations, including young children, older adults, and individuals with comorbidities [8]. In this regard, the rapid, specific, and simultaneous detection of respiratory pathogens is a key strategy to guide clinical management and support control efforts [9]. Improved timeliness of diagnosis and multiplex detectability can also contribute to reducing antibiotic use [10], limiting the need for additional laboratory tests [11], and optimizing cost-effectiveness [12].

Although multiple diagnostic approaches exist, nucleic acid amplification tests (NAATs)—particularly multiplex RT-qPCR—are considered the clinical gold standard due to their sensitivity and specificity and their ability to detect multiple targets in a single reaction [13,14,15,16,17,18]. In 2021, the U.S. Centers for Disease Control and Prevention (CDC) introduced the CDC Flu-SC2 assay for simultaneous detection of influenza A, influenza B, and SC2 [19]; however, it does not include RSV, and its semi-automated workflow requires manual handling. This study aimed (i) to develop an RSV A/B RT-qPCR assay (LDT RSV) compatible with CDC Flu-SC2 chemistry, and (ii) to validate a fully automated laboratory-developed respiratory assay (LDRA) on the Panther Fusion^®^ Open Access™ system for simultaneous detection of influenza A, influenza B, SC2, and RSV in nasopharyngeal swabs from patients with suspected respiratory infection.

## 2. Materials and Methods

### 2.1. Clinical Samples, Ethical Aspects, and Eligibility

This single-center study was conducted by the Research and Development (R&D) Group of the Molecular Biology Department at Referencia Laboratorio Clínico (Santo Domingo Oeste, Dominican Republic). We used residual material from 405 nasopharyngeal swabs collected in Universal Transport Medium (UTM; Copan, CA, USA) from patients with clinician-ordered molecular testing for respiratory infection by IAV, IBV, SC2, and/or RSV. Specimens were randomly selected between October 2023 and September 2025. In accordance with laboratory confidentiality and informed-consent policies, all patients consented to research use of residual specimens and publication of results with privacy safeguards; accordingly, all samples were de-identified prior to analysis.

Pre-analytical acceptance/rejection criteria were applied according to the laboratory standard operating procedures (SOPs) for respiratory NAAT testing. Specimens were rejected if they: (i) were mislabeled or had mismatched identifiers; (ii) were collected in an inappropriate tube/transport medium; (iii) were in a leaking or broken container; (iv) had insufficient specimen volume; (v) were stored or transported outside SOP specifications; (vi) had gross specimen quality issues likely to compromise NAAT performance (e.g., obvious contamination); or (vii) had no or invalid test prescription. All residual clinical samples included in this evaluation satisfied the laboratory’s acceptance criteria. No demographic eligibility criteria were applied. Residual specimens were stored at −70 °C immediately after routine testing.

### 2.2. Assay Design and Optimization

#### 2.2.1. Oligo Design

**Figure 1:**
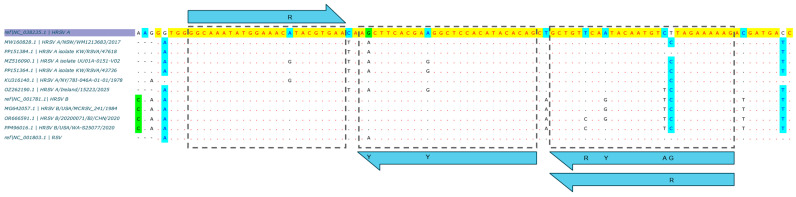
Target sequence, primers and probe of the LDT RSV: amplicon, 80 bp.

Oligonucleotides for LDT RSV were designed as previously described [20,21]. In brief, 8661 complete RSV sequences (types A and B) were retrieved from the NCBI Virus database (https://www.ncbi.nlm.nih.gov/labs/virus/, accessed on 30 September 2025) and aligned with MAFFT algorithm in Unipro UGENE v48.0 (Unipro, Novosibirsk, Russia). After alignment validation, redundancy removal, and reduction to 8100 sequences, a conserved region within the RSV *Matrix* (*M*) gene was selected by sequence-entropy analysis (Supplementary Figures S1 and S2, based on Supplementary Files S1–S4). Using the alignment-derived consensus sequence as reference, three primers and one TaqMan probe were designed employing Oligo v7.60 (Molecular Biology Insights Inc., Colorado Springs, CO, USA), Benchling™ (Benchling, San Francisco, CA, USA), and OligoAnalyzer™ (Integrated DNA Technologies, Coralville, IA, USA) (Figure 1). The probe was labeled at the 5′ end with cyanine 5 (Cy5) fluorophore, phosphorylated and labeled at the 3′ end with the Iowa Black^®^ RQ dark quencher (IAbRQSp), and contained an internal TAO™ quencher at the ninth nucleotide (5′→3′). The LDT RSV oligos were designed to minimize heterodimer formation with the CDC Flu-SC2 oligos [19]. To improve resilience to mutations in circulating SC2 variants, the CDC SARS-CoV-2 detection probe was modified. All oligos comprising the quadruplex LDRA assay (CDC Flu-SC2 + LDT RSV) are provided in Table 1.

All oligonucleotides were synthesized and purified by HPLC by Integrated DNA Technologies (IDT, Coralville, IA, USA). Optimal purity was defined as ≥85%. All oligonucleotides were shipped lyophilized.

#### 2.2.2. In Silico Specificity Evaluation

In silico specificity of the RSV primers and probe was assessed as previously described [21] by evaluating homology, exclusivity (cross-reactivity), and potential amplification of human background DNA. Homology was assessed by open NCBI BLAST tool searches (https://blast.ncbi.nlm.nih.gov, accessed on 30 September 2025). For 100% coverage, high-homology sequences were defined as ≥95% identity and an expectation value (*E*-value) ≤ 10^−2^ [23]. Exclusivity was assessed by closed searches against sequences from ≥68 clinically and biologically relevant respiratory microorganisms (Supplementary Table S1); low homology (and thus low cross-reactivity likelihood) was defined as *E*-value > 10^−2^ irrespective of coverage or identity [21].

Potential amplification of human DNA was evaluated with MFEprimer version 3.1 [24] (https://mfeprimer3.igenetech.com/ or https://m4.igenetech.com/, accessed on 30 September 2025). The following parameters were set: “Background Database”, “UCSC–Homo sapiens–hg38–Genome”; “Allow mismatch at the 3′ end”, “No” (default); “Tm min”, “48 °C” (minimum Tm required for binding stability between the primer and its binding sites: annealing temperature (T_a_) minus10 °C); “Concentration of divalent cations (usually MgCl_2_)”, “4.0 mM”; “Annealing oligo concentration”, “800 nM” (optimized primer concentration); “Product size”, from “0” to “1000” bp.

Inclusivity was estimated by design-coverage analysis using the SCREENED v1.0 tool [25] (Sciensano Galaxy External; https://galaxy.sciensano.be/, accessed on 30 September 2025) with all sequences available in the NCBI Virus (https://www.ncbi.nlm.nih.gov/labs/virus/, accessed on 30 September 2025) and GISAID EpiRSV™ (https://gisaid.org/, accessed on 30 September 2025): 3705 RSV A and 3178 RSV B sequences from NCBI Virus, and 11,752 RSV A and 13,910 RSV B sequences from GISAID. Sequences with missing or ambiguous information were removed.

#### 2.2.3. Synthetic Controls: Ultramer™ Duplex

Synthetic Ultramer™ duplex oligonucleotides (≤200 bp) containing the target regions for IAV, IBV, SC2, RSV A, and RSV B were used as amplification controls during assay optimization (Supplementary Table S2). Controls were synthesized and shipped lyophilized (4 nmol) by Integrated DNA Technologies (IDT, Coralville, CA, USA) after standard desalting.

#### 2.2.4. Ultramer™ Duplex Control Preparation: Stock Solution

Ultramer™ duplex controls were reconstituted and stock solutions prepared as previously described [21]. Briefly, each 4 nmol control was reconstituted in 1 mL IDTE buffer (10 mM Tris, 0.1 mM EDTA), pH 8.0 (IDT, Coralville, IA, USA). Thirteen additional 1:10 serial dilutions were prepared in the same buffer, yielding 14 concentration levels per control (stock solution panel: 2.41 × 10^15^ to 2.41 × 10^2^ copies/mL).

#### 2.2.5. Assay Optimization

Physicochemical conditions for each monoplex assay composing the quadruplex LDRA were optimized as previously described [20,21]. Salt concentrations (KCl and MgCl_2_) were optimized first, followed by primers/probe concentrations. PCR-grade betaine (5 M; Sigma-Aldrich, St. Louis, MO, USA) and annealing temperature were then optimized using a 10 °C gradient (54–64 °C: 54 °C, 54.7 °C, 56 °C, 57.9 °C, 60.4 °C, 62.3 °C, 63.5 °C and 64 °C). All other cycling parameters were kept at the default Panther Fusion^®^ Open Access™ RNA amplification standard protocol so that the critical amplification time (55 min) was not exceeded.

Primer and probe reconstitution solutions (PPR) for optimization experiments were prepared in 8 mM Tris-HCl buffer. Optimization runs used the off-label Panther Fusion^®^ Open Access™ RNA/DNA Enzyme Cartridge (Hologic, Inc., Marlborough, MA, USA) protocol on a CFX96-IVD thermal cycler (Bio-Rad, Hercules, CA, USA) and Ultramer™ duplex controls at a nominal concentration of 2.41 × 10^5^ copies/mL (Ct ≈ 30). The optimized thermocycling program was: 1 cycle at 46 °C for 8 min, 1 cycle at 95 °C for 2 min, and 45 cycles of 95 °C for 5 s and 58 °C for 21 s (fluorescence read). MyAccess™ Software v2.1.2.1 (Hologic, Inc., Marlborough, MA, USA) was used to implement the LDT protocol on the Panther Fusion^®^ system; the final Open Access™ protocol is provided in Supplementary File S5.

Supplementary Table S3 summarizes the optimized PPR composition for the quadruplex LDRA.

#### 2.2.6. Amplification Efficiency and Multiplex Compatibility

Amplification efficiency (*E*) and multiplex compatibility were evaluated post-optimization as previously described [21]. Ten-fold serial dilutions (2.41 × 10^9^ to 2.41 × 10^2^ copies/mL) of each synthetic Ultramer™ duplex control were tested in triplicate. The fluorescence threshold was set at approximately the midpoint of the linear phase of the amplification curves. Standard curves were generated by plotting cycle threshold (Ct) values versus log_10_ (input concentration; C_i_) using Microsoft^®^ Excel (Microsoft Corp., Redmond, WA, USA), and *E* and its 95% confidence intervals (95% CI) were calculated from the regression slope (m) using the following equation.

(1) *E* = (10^−1/m^ − 1) × 100%.

*E* was considered acceptable for values between 90% and 110% with goodness of fit (R^2^) ≥ 0.98.

For multiplex compatibility, Ct values and *E* between monoplex and multiplex conditions were computed. Multiplex compatibility was considered acceptable when each monoplex–multiplex pair: (i) the |ΔCt| ≤ 0.5; (ii) the *E* acceptability criterion described above was satisfied; and (iii) no indications of significantly compromised sensitivity in the multiplex reaction (e.g., failure or inefficient amplification) were observed.

For these purposes, the off-label protocol of the Panther Fusion^®^ Open Access™ RNA/DNA Enzyme Cartridge was used on the CFX96-IVD analyzer.

The *E* per target was verified under the definitive conditions of the quadruplex LDRA assay on the Panther Fusion^®^ Open Access™ system.

#### 2.2.7. Panther Fusion^®^ Open Access™ RNA/DNA Enzyme Cartridge: Off-Label Protocol

Briefly, the wells of the Panther Fusion^®^ Open Access™ RNA/DNA Enzyme Cartridge containing the enzyme aliquots were punctured using a nuclease-free punching tool. The lyophilized enzyme was reconstituted with 20 μL of PPR, incubated at room temperature (18–25 °C) for 1–5 min, and mixed by vortexing in short, repeated pulses to minimize foaming. Amplification and detection were performed on the CFX96-IVD analyzer using 96-well PCR plates in a 25 μL reaction volume (20 μL master mix (MMX) + 5 μL DNA template).

### 2.3. Analytical Performance

#### 2.3.1. Analytical Sensitivity (Limit of Detection)

The limit of detection (LoD) was estimated using dilution panels prepared from the five synthetic Ultramer™ duplex controls. Starting from stock solutions at 2.41 × 10^7^ copies/mL, each control was first diluted to a working concentration of 6.4 × 10^3^ copies/mL in a UTM/Panther Fusion^®^ Specimen Lysis Tube (SLM) medium matrix. UTM from negative nasopharyngeal swabs was mixed with SLM (Hologic Inc., Marlborough, MA, USA) at the manufacturer-recommended proportion (0.5 mL UTM per 0.71 mL SLM) [26] to generate the UTM/SLM.

Each LoD panel consisted of seven additional 1:2 serial dilutions prepared from the working solution in UTM/SLM (final volume, 1000 µL), yielding eight 1:2 dilution levels ranging from 6.4 × 10^3^ copies/mL (2.3 × 10^2^ copies/reaction) to 5.0 × 10^1^ copies/mL (1.8 × 10^0^ copies/reaction). These concentrations correspond to 1.5 × 10^4^ to 11.2 × 10^2^ copies/mL in the primary UTM specimen (prior to mixing with SLM).

Copies/mL in UTM/SLM were converted to copies/reaction using the equation shown, assuming: 360 µL sample aspirated, 50 µL elution volume, 5 µL eluate per amplification reaction, 100% extraction recovery, and 1000 as the conversion factor from mL to µL.

(2) C(PCR) [copies/reaction] = C(UTM/SLM) [copies/mL]/1000 × 360/50 × 5 C(PCR) [copies/reaction] = C(UTM/SLM) [copies/mL] × 0.036

where C(PCR) [copies/reaction] is the number of copies of the target sequence in the PCR reaction; C(UTM/SLM) [copies/mL] is the concentration of the target sequence, in copies/mL, in the UTM/SLM.

Equivalent concentrations in the primary UTM specimen were calculated by volumetry using the stated mixture volumes (V(UTM/SLM) = 1.21 mL; V(UTM) = 0.5 mL).

(3) C(UTM) [copies/mL] = C(UTM/SLM) [copies/mL] × V(UTM/SLM) [mL]/V(UTM) [mL] C(UTM) [copies/mL] = C(UTM/SLM) [copies/mL] × 1.21 mL/0.5 mL C(UTM) [copies/mL] = C(UTM/SLM) [copies/mL] × 2.42

where C(UTM) [copies/mL] is the concentration, in copies/mL, of the target sequence in the primary UTM specimen; C(UTM/SLM) [copies/mL] is the concentration of the target sequence, in copies/mL, in the UTM/SLM.

Using a single lot of specific (PPR) and generic reagents, a single instrument, and one operator, 20 replicates of each member of the evaluation panels were analyzed, and the frequency of positive results was recorded at each concentration level. C_95_ LoD and their 95% CI estimates were obtained by probit regression of detection probability as a function of concentration. The cutoff was set at the maximum possible threshold cycle of the employed amplification and detection protocol (Ct = 45).

#### 2.3.2. Analytical Specificity: Cross-Reactivity (In Vitro Exclusivity) and In Vitro Inclusivity

Analytical specificity was evaluated using pathogen panels assembled from commercial quality-control materials and external quality assessment (EQA) schemes. Commercial controls/panels included NATtrol Respiratory Verification Panel 2.1 (ZeptoMetrix, Buffalo, NY, USA), Respiratory Multiplex (1 to 5) Q Control (Qnostics Ltd., Glasgow, Scotland, UK), Amplirun^®^ SARS-CoV-2 RNA Controls (Amplirun^®^ SARS-CoV-2 B.1.1.7 (Alpha) RNA Control, Amplirun^®^ SARS-CoV-2 B.1.351 (Beta) RNA Control, Amplirun^®^ SARS-CoV-2 B.1.617.2 (Delta) RNA Control, Amplirun^®^ SARS-CoV-2 P.1 (Gamma) RNA Control, Amplirun^®^ SARS-CoV-2 BA.1 (Omicron) RNA Control) (Vircell S.L., Granada, Andalucía, Spain), Amplirun^®^ Total SARS-CoV-2/FluA/FluB/RSV Control (Swab) (Vircell S.L., Granada, Andalucía, Spain), and AccuPlex™ H5N1 Influenza Reference Material Kit (LGC Clinical Diagnostics Inc., Milford, MA, USA). EQA materials were from CAP ID3 and IDR (CAP: College of American Pathologists; 2023 and 2024) and QCMD INFTP24 (QCMD: Quality Control for Molecular Diagnostics; 2024). Each panel member was tested in triplicate with the LDRA assay.

For cross-reactivity assessment, 30 respiratory pathogens were tested at clinically relevant concentrations (per manufacturer specifications): Adenovirus A, Adenovirus 1, Adenovirus 3, Adenovirus 14, Adenovirus 31, *Bordetella parapertussis*, *Bordetella pertussis*, *Chlamydia pneumoniae*, Coronavirus 229E, Coronavirus HKU-1, Coronavirus NL63, Coronavirus OC43, Enterovirus A16, Enterovirus 68, Enterovirus A71, Influenza A, Influenza B, *Mycoplasma pneumoniae*, Metapneumovirus 8, Metapneumovirus A2, Metapneumovirus B2, Parainfluenza 1, Parainfluenza 2, Parainfluenza 3, Parainfluenza 4, Rhinovirus 1A, Rhinovirus 16, RSV A, SARS-CoV-2, and *Legionella pneumophila*.

For in vitro inclusivity, 32 distinct (no duplicate) variants/strains were tested: (i) 17 IAV variants/strains, including seasonal H1N1 (H1N1sea), 2009 pandemic-derived H1N1 (H1N1pdm09), H3N2, H5N1 and H7N7 subtypes; (ii) four IBV variants/strains spanning Victoria and Yamagata lineages; (iii) seven SC2 variants/strains including Alpha, Beta, Gamma, Delta and Omicron lineages; and (iv) four RSV variants/strains spanning subtypes A and B.

#### 2.3.3. Precision

Within-laboratory precision of the quadruplex LDRA assay was assessed using a five-member panel prepared in UTM/SLM. The panel included one negative matrix member and four doubly positive members consisting of IAV + SC2 and IBV + RSV at two concentration levels: low (30 < Ct ≤ 35) and moderate (25 < Ct ≤ 30). Only low–moderate and moderate–low pairings were evaluated for each pathogen combination. Panel pathogens were IAV, A/NY/02/09 (H1N1pdm09); IBV, B/Florida/02/06 (Victoria); SC2, USA-WA1/2020; and RSV type A, all from NATtrol Respiratory Verification Panel 2.1.

Each panel member was tested in duplicate by a single operator in one daily run on two Panther Fusion^®^ systems, using two PPR lots, over 14 consecutive days. Panel samples were stored at 2 °C to 8 °C throughout the evaluation, and a new PPR vial from each lot was thawed daily.

Agreement with expected results was calculated for each pathogen and panel member. Within-laboratory precision, and its components (within-lot, between-lot, between-instrument, within-run/day [repeatability], and between-run/day precision), were summarized as standard deviation (SD) and coefficient of variation (CV).

#### 2.3.4. Method Comparison: Agreement Assessment

Agreement was assessed using 405 clinical samples tested with the quadruplex LDRA assay and two commercial comparators: Panther Fusion^®^ SARS-CoV-2/Flu A/B/RSV Assay (PFRA; Hologic, Inc., Marlborough, MA, USA) and Allplex™ SARS-CoV-2/FluA/FluB/RSV Assay (APRA; Seegene Inc., Seoul, Republic of Korea). Discordant specimens were retested with all assays. Persistently discordant results were adjudicated using the consensus rules described below (see Section 2.4). Specimens yielding an “invalid” result were excluded.

##### Allplex™ SARS-CoV-2/FluA/FluB/RSV Assay (APRA)

Clinical samples were tested with APRA, a multiplex RT-qPCR assay for simultaneous detection of SC2, IAV, IBV and RSV A/B. Viral RNA was extracted with the MagNA Pure 96 DNA and Viral NA Large Volume kit (Roche Molecular Systems, Inc., Branchburg, NJ, USA) on the MagNA Pure 96 instrument using the “Viral NA Universal LV 4.0” protocol (500 µL input; 50 µL elution), following the manufacturer’s instructions [27]. Amplification and detection were performed on the CFX96-IVD analyzer [28] using kit-provided enzyme mixes and oligonucleotides in a 20 µL reaction volume. The assay includes endogenous (Endo IC) and exogenous (Exo IC) internal controls.

APRA targets the SC2 *N*, *RdRp*, and *S* genes; the IAV *M* gene; the IBV *NS2* gene; and the RSV *M* gene. Results were interpreted with Seegene Viewer v3.33 (Seegene Inc., Seoul, Republic of Korea) using channels FAM (SC2 *S* gene and RSV), HEX (SC2 *RdRp* gene and IBV), Cal Red 610 (SC2 *N* gene and IAV), and Quasar 670 (Endo and Exo IC). Total time from extraction to results was approximately 4 h.

##### LDRA and Panther Fusion^®^ SARS-CoV-2/Flu A/B/RSV Assay (PFRA)

All clinical samples and analytical-specificity panel specimens were stabilized in SLM according to the manufacturer’s instructions [26] and stored at 2–8 °C for ≤10 days. Stabilized specimens were tested with both the candidate LDRA and the comparator PFRA per manufacturer specifications [29].

PFRA is supplied as lyophilized, ready-to-use reagents in cartridges (up to 12 tests per cartridge; up to 96 tests per box), whereas LDRA uses a user-prepared, target-specific PPR together with the generic Panther Fusion^®^ Open Access™ RNA/DNA Enzyme Cartridge. By default, the Panther Fusion^®^ extraction protocol aspirates 360 µL of stabilized specimen and elutes nucleic acids in 50 µL. RT-qPCR amplification/detection for both assays occurs in the Fusion^®^ module with a 25 µL reaction volume (20 µL MMX + 5 µL eluate). Both assays used Panther Fusion^®^ Extraction Reagent-S and Internal Control-S (IC-S; Hologic, Inc., Marlborough, MA, USA), although IC-S is reported only by PFRA.

PFRA targets the IAV, IBV and RSV M genes and the SC2 Orf1ab gene. LDRA targets the IAV and RSV M genes, the IBV NS2 gene and the SC2 N gene. The Panther Fusion^®^ system interprets fluorescence in channels FAM (IAV in both assays), HEX (IBV in LDRA; RSV in PFRA), ROX (SC2 in both assays), Quasar 670 (RSV in LDRA; IBV in PFRA), and Quasar 705 (internal control (IC): human RNase P for LDRA). PFRA interpretation is proprietary; LDRA was configured as: “positive” when the sigmoidal fluorescence curve crossed the predefined threshold (200 Relative Fluorescence Units (RFU) for IAV and IBV; 100 RFU for SC2; 50 RFU for RSV; 100 RFU for RNase P), “negative” when the threshold was not crossed and the IC was positive, and “invalid” when all channels (including the IC) were negative.

Turnaround time from extraction to results on the Panther Fusion^®^ system was approximately 2.5 h for both assays.

### 2.4. Diagnostic Performance

Diagnostic sensitivity (dSens), diagnostic specificity (dSpec), positive predictive value (PPV), and negative predictive value (NPV) for LDRA, PFRA and APRA were calculated using 2 × 2 contingency tables against an expected result defined as the per-target consensus across the three assays. Consensus rules were: (A) “positive” if at least two of the three assays were positive for the same target; and (B) “negative” if at least two of the three assays were negative.

### 2.5. Mixed Infection Detection Capability

Agreement for mixed-infection detection was assessed using only samples positive for any of IAV, IBV, SC2, or RSV (*n* = 335). A consensus reference was defined as: (A) “mixed infection” (Mix) if at least two of three assays detected the same ≥ 2 pathogens in the same specimen; and (B) “single infection” (Sin) if at least two of three assays detected the same single pathogen only.

### 2.6. Statistical Analysis

Slopes (Ct vs. log_10_(C_i_)) and amplification efficiencies (per target) for LDRA were estimated by linear regression in Microsoft^®^ Excel; 95% CI were computed from the standard error of the slope.

For precision, Grubbs’ test was applied to identify outlier Ct values. After outlier exclusion, a three-way ANOVA was used to estimate within-laboratory precision components (within-lot, between-lot, between-instrument, between-run/day, and within-run/day precision), summarized as SD and CV.

Agreement for target detection and mixed-infection detection capability among LDRA, PFRA, and APRA was assessed using 2 × 2 contingency tables to compute positive/mixed-infection percent agreement (PPA/MixPA), negative/single-infection percent agreement (NPA/SinPA), overall percent agreement (OPA), and Cohen’s *kappa* (*κ*-index). Excellent agreement was defined as OPA ≥ 95% and *κ* > 0.8 [30,31].

Wilson score intervals were used to compute 95% CI for agreement estimates (PPA/MixPA, NPA/SinPA, OPA), positivity rates (PR), and diagnostic performance metrics (dSens, dSpec, PPV, NPV). The 95% CI for *κ*-index and SD were calculated using Wald for binomial proportions and Exact/MLS methods, respectively [32].

The McNemar-Mosteller nonparametric test was used to assess the statistical significance of differences in PR, dSens, and dSpec between the candidate and comparator assays (LDRA, PFRA, and APRA).

Analyses were performed with Analyse-it^®^ for Microsoft^®^ Excel, Ultimate Edition v6.16.2 (Analyse-it Software Ltd., Leeds, UK). A statistical significance level of 5% (*p* < 0.05) was considered.

## 3. Results

### 3.1. Design Evaluation and Assay Optimization

#### 3.1.1. In Silico Specificity

BLAST (https://blast.ncbi.nlm.nih.gov) analysis of the RSV primers/probe showed that sequences meeting the predefined high-homology criteria (coverage and percent identity ≥ 95% with *E*-value ≤ 10^−2^) were observed only for RSV (Supplementary File S6). In the closed-search exclusivity panel, all 68 respiratory-related microorganisms yielded coverage/identity < 95% and *E*-value > 10^−2^, corresponding to 100% in silico exclusivity (Supplementary File S7). MFEprimer v3.1 predicted negligible amplification of human background DNA by the RSV oligos (Supplementary File S8).

A comparison with analogous oligo designs previously published can be found in Supplementary Figures S3 and S4.

Inclusivity analysis of the LDT RSV oligos yielded predicted true-positive rates of 99.73% and 99.93% across 3360 RSV A and 2834 RSV B NCBI sequences, respectively. Consistently, analysis of 11,653 RSV A and 13,907 RSV B sequences from GISAID estimated inclusivity of 99.06% and 99.89%, respectively (Supplementary Table S4, based on Supplementary Files S9–S12). When NCBI and GISAID datasets were pooled, overall inclusivity (pooled inclusivity) reached 99.21% for RSV A and 99.9% for RSV B (Supplementary Figure S4).

#### 3.1.2. Amplification Efficiency and Multiplex Compatibility

**Figure 2:**
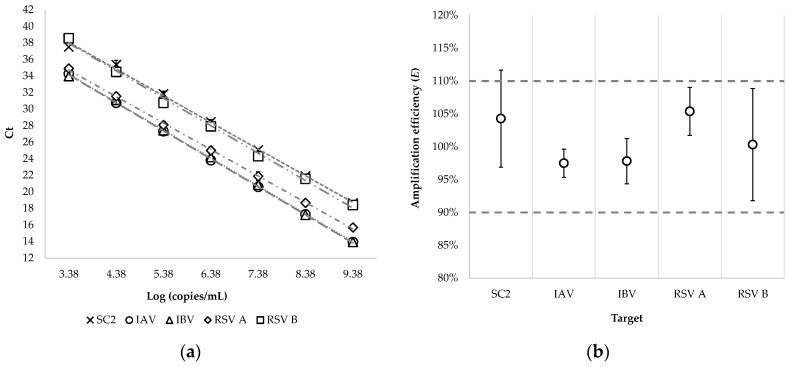
Amplification efficiency analysis per target in a multiplex setting (LDRA): (**a**) Linear regression per assay, with error bars (standard deviation) per level; (**b**) amplification efficiency (*E*) per target, with its 95% CI and acceptance limits (dashed lines). IAV, influenza A virus; IBV, influenza B virus; SC2, SARS-CoV-2; RSV A, respiratory syncytial virus type A; RSV B, respiratory syncytial virus type B.

Monoplex assays met the predefined amplification-efficiency acceptability range (90% ≤ *E* ≤ 110%), and on the Panther Fusion^®^ Open Access™ system the LDRA achieved R^2^ ≥ 0.99 with all targets remaining within the predefined efficiency limits (Table 2, Figure 2, and Supplementary Figure S5).

### 3.2. Analytical Performance

#### 3.2.1. Analytical Sensitivity (Limit of Detection)

Absolute and relative hit frequencies by target and dilution level are shown in Supplementary Table S5.

According to the probit regression, the LDRA C_95_ LoDs were: 37.8 copies/reaction (95% CI: 29.5–58.0) for IAV; 13.9 copies/reaction (95% CI: 11.0–21.1) for IBV; 9.6 copies/reaction (95% CI: 7.4–14.6) for SC2; 12.3 copies/reaction (95% CI: 9.6–18.0) for RSV A; and 25.1 copies/reaction (95% CI: 20.4–34.4) for RSV B (Table 3 and Supplementary Figure S6).

#### 3.2.2. Analytical Specificity

##### Cross-Reactivity (In Vitro Exclusivity)

No cross-reactivity was observed with any pathogens included in the analytical-specificity panel. Sigmoidal amplification curves were observed only in the channels corresponding to IAV, IBV, SC2, and RSV (Table 4).

##### In Vitro Inclusivity

All subtypes and variants of the respiratory viruses tested with LDRA produced “positive” results in the expected detection channels for IAV, IBV, SC2, and RSV; no false-negative results were found in the in vitro inclusivity panel (Table 5).

#### 3.2.3. Precision

**Figure 3:**
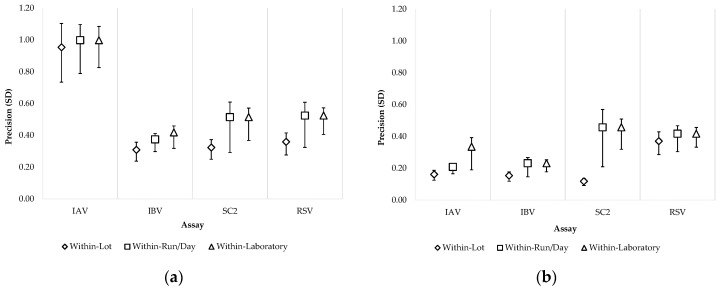
Laboratory precision of the quadruplex LDRA: (**a**) Low level; (**b**) Moderate level. The diamond represents the within-lot precision, the square represents the within-run/day precision, and the triangle represents the within-laboratory precision. The error bars represent the 95% CI. IAV, influenza A virus; IBV, influenza B virus; SC2, SARS-CoV-2; RSV, respiratory syncytial virus.

Two outlier Ct values and two invalid results due to instrument errors were excluded. After exclusions, agreement with expected results exceeded 99%. Within-laboratory precision for the moderate level was 0.33 (95% CI: 0.28–0.48), 0.23 (0.21–0.29), 0.46 (0.40–0.59), and 0.42 (0.38–0.50) for IAV, IBV, SC2 and RSV, respectively; and for the low level was 1.00 (0.91–1.17), 0.42 (0.38–0.52), 0.51 (0.46–0.66), and 0.52 (0.48–0.64) for IAV, IBV, SC2 and RSV, respectively (Table 6). Figure 3 summarizes the main components contributing to within-laboratory precision of the LDRA assay.

#### 3.2.4. Method Comparison: Agreement Assessment

No invalid results were found with any assay during method comparison; therefore, no specimens were excluded. Relative to PFRA, four SC2 specimens and one RSV specimen were PFRA-positive/LDRA-negative (0.99% and 0.25%, respectively; 1.23% overall). Relative to APRA, one specimen each for IAV, IBV, and RSV was LDRA-positive/APRA-negative (0.25% each; 0.74% overall). Discordant specimens were reanalyzed with all three RT-qPCR assays, and the same results were reproduced. One specimen was ultimately considered SC2-positive by all three assays after testing six replicates, with hit rates of 2/6 (LDRA), 3/6 (PFRA), and 6/6 (APRA); APRA reactivity was observed only for the SC2 *S* gene across all replicates. Across both comparator pairs, agreement metrics for detection of the four viruses were: NPA ≥ 99.7% (95% CI: 98.1–99.9%), PPA ≥ 95.7% (95% CI: 89.3–98.3%), OPA ≥ 99.0% (95% CI: 97.5–99.6%), and *κ* ≥ 0.971 (95% CI: 0.944–0.999) (Table 7).

Discordances between LDRA and PFRA were associated with high Ct values (SC2: 37.3–39.7; RSV: 38.8). Likewise, discordances between LDRA and APRA occurred at similarly high Ct values (IAV: 36.6; IBV: 37.6; RSV: 38.0). Consistent with these low-template patterns, the specimen that showed variable detection across six SC2 replicates yielded comparable mean Ct values across assays: LDRA, 37.8 (2/6 positive replicates); PFRA, 38.3 (3/6); and APRA, 34.2 (6/6).

### 3.3. Diagnostic Performance

According to the McNemar–Mosteller nonparametric test, positivity rates (PR) for IAV, IBV, SC2 and RSV did not differ significantly between LDRA and the comparator assays PFRA and APRA (*p* > 0.7322). Using the per-target consensus reference (majority agreement among the three assays) (see Section 2.4), LDRA showed 100% dSens and dSpec for all targets (Table 8). PFRA yielded four SC2 false-positive results and one RSV false-positive result, whereas APRA yielded one false-negative result each for IAV, IBV and RSV; however, no statistically significant differences in dSens or dSpec were observed among assays (*p* > 0.1250).

### 3.4. Mixed Infection Detection Capability

Among 335 specimens positive for ≥1 target, seven (2.09%) showed consensus evidence of mixed infection: one SC2 + IBV, one IBV + RSV, one IAV + RSV, three SC2 + RSV, and one SC2 + IAV. The LDRA assay detected all seven co-infections with no disagreement versus the consensus results (OPA = 100% [95% CI: 98.9–100%]; *κ* = 1.000). LDRA detected all seven co-infections without disagreement versus the consensus (OPA = 100% [95% CI: 98.9–100%]; *κ* = 1.000). PFRA detected the seven consensus co-infections plus three additional discordant co-infections (one IAV + RSV and two SC2 + IAV; OPA = 99.1% [95% CI: 97.4–99.7%]; *κ* = 0.819 [95% CI: 0.619–1.000]). APRA detected five of the seven consensus co-infections; one specimen was called SC2 only (instead of SC2 + IBV) and another IAV only (instead of IAV + RSV) (OPA = 99.4% [95% CI: 97.8–99.8%]; *κ* = 0.830 [95% CI: 0.599–1.000]) (Table 9).

## 4. Discussion

This work designs an oligo set for detection of RSV A/B and validates a laboratory-developed respiratory assay (LDRA), resulting from combining the LDT RSV with the CDC Flu-SC2 [19]. The new quadruplex enables fully automated, multiplex RT-qPCR detection of IAV, IBV, SC2, and RSV on the Panther Fusion^®^ Open Access™ platform. In the current post-pandemic setting—where influenza, RSV, and SC2 can co-circulate and produce clinically indistinguishable syndromes [2,33]—such syndromic molecular testing can improve time-to-diagnosis and strengthen infection control and surveillance workflows [12]. In our proposal, using of human RNase P as an IC instead of IC-S adds a safety element because it ensures adequate sample collection. Assays that rely only on process controls, such as the PFRA comparator used in this study, introduce an additional risk of false negatives when clinical samples have not been collected effectively [34].

Although a commercial assay is available for the Panther Fusion^®^ platform (PFRA), we validated our approach using the system’s open channel because of local operational availability and the need to implement a resource-optimized workflow while maintaining equivalent quality standards. Laboratory-developed tests (LDTs) have been recognized as an important tool for laboratories seeking to adapt diagnostic capacity to local needs [35,36,37]. In settings where health insurance coverage can be fragmented or insufficient, as is often the case in the Dominican Republic, LDTs may facilitate more equitable access to molecular diagnostics, which remain relatively costly.

From an operational perspective, the Panther Fusion^®^ platform provides mid-to-high throughput with continuous loading and random access, which can support timely reporting during seasonal peaks. Increased automation across pre-analytical to post-analytical steps reduces manual handling and may decrease the likelihood of human error, thereby improving reproducibility and supporting scalability in routine clinical workflows.

Analytically, the quadruplex LDRA demonstrated consistent performance in multiplex format, with amplification efficiencies within predefined acceptance limits and no evidence of target interference. The RSV component also showed high predicted inclusivity in silico across large RSV A and B sequence datasets, supporting resilience to circulating genetic diversity, and in vitro specificity testing against a comprehensive respiratory pathogen panel showed no cross-reactivity. Likewise, the remaining oligo designs derived from CDC Flu-SC2 for IAV, IBV, and SC2 detection (including the SC2 probe modification described here) retained high analytical specificity in silico (Supplementary Table S6) and in vitro (Table 4 and Table 5).

A dedicated analytical sensitivity study was performed for each target using standardized replicate testing and probit analysis. The LDRA assay achieved C_95_ LoDs in the low tens of copies per reaction, corresponding to approximately 10^3^–10^4^ copies/mL, depending on the target. Direct LoD comparisons across candidate (LDRA) and comparator (PFRA and APRA) assays are inherently constrained by differences in sample materials, matrices, and reporting units. However, compared with the CDC Flu-SC2 assay, the quadruplex configuration showed slightly higher LoDs for influenza and SC2 (9.6–37.8 copies/reaction vs. 5 copies/reaction) [19]. This difference may reflect the lower effective input volume (approx. 15 µL) dictated by the platform workflow. Overall, the LoDs observed for all four targets align with analytical sensitivity benchmarks commonly reported for contemporary multiplex RT-qPCR assays, particularly when assays are capable of detecting ≤ 10^2^ copies/reaction (≤10^4^ copies/mL) [3,5,19,38,39,40,41].

The main contributors to within-laboratory imprecision for the LDRA assay were run/day (within-run/day precision or repeatability) and PPR lot (within-lot precision). Instrument effects (between-instrument precision) contributed to a lesser extent, depending on the pathogen and concentration level. For RT-qPCR assays, CV < 10% and CV < 5% are generally considered good and excellent, respectively [42]. Accordingly, the per-target precision metrics reported here for LDRA (CV: 0.9–2.9%; SD: 0.23–1.00) indicate high precision and demonstrate consistency, even near the LoD. These findings are also consistent with Shu et al. [19], who considered variance values > 2 (SD = 1.41) unacceptable during CDC Flu-SC2 production quality control. Prior reports are consistent with these criteria [7,10,40], including manufacturer-reported precision for the commercial comparator assays (PFRA and APRA). For PFRA, a maximum reported CV of 2.04% was reported for a low level of IAV [29]. For APRA, a CV of less than 10% was considered acceptable [28].

The LDRA assay showed very high agreement with both commercial comparators across a broad Ct range, with percent agreement and *κ* (*kappa*) coefficient approaching 100% and only a small number of discordant results. Recent studies likewise report substantial inter-assay commutability and agreement among multiplex RT-qPCR assays for simultaneous detection of IAV, IBV, SC2, and RSV, with percent agreement values between 95% and 100% [3,5,6,7,8,16,39,43]. Although comparative statistics in the literature may vary across study designs and specimen sets, RT-qPCR assays for diagnosing IAV, IBV, SC2, and RSV generally meet commonly accepted acceptability criteria [30,31]. Against the study’s consensus reference standard (majority agreement among the three assays), LDRA showed 100% dSens and dSpec for each target. Importantly, because the consensus is derived from the three assays, LDRA’s “100% accuracy” should be interpreted as complete agreement with the majority-rule consensus rather than as proof of infallibility against an independent gold standard. Nevertheless, the concordance pattern—together with repeat testing of discordant specimens—supports that LDRA’s diagnostic performance is comparable to that of established commercial systems. No statistically significant differences were detected across assays (*p* > 0.1250), consistent with previous studies reporting diagnostic sensitivities and specificities that typically range from 95% to 100% and from 96.8% to 100%, respectively [12,17,18,44].

Discordant results in the comparator assays clustered at high Ct values, consistent with low-titer specimens in which stochastic sampling near the LoD can yield intermittent detection and increase the likelihood of apparent false negatives on repeat aliquots. In addition, multiplex assays may differ in target selection, chemistry, and signal processing; minor cross-channel fluorescence bleed-through (“crosstalk”) or differences in thresholding algorithms could contribute to occasional false-positive calls on specific platforms. In this dataset, the pattern of PFRA false positives (notably for SC2 and RSV) and APRA false negatives (one each for IAV, IBV, and RSV) is consistent with rare, low-frequency events expected near decision thresholds rather than with systematic assay failure, supported by the reproducibility of discordant calls upon reanalysis and by the observation that at least one SC2 specimen showed variable hit rates across replicate testing. Notably, SC2 false positives with PFRA—the most frequent discordance in this study—have been previously reported [16]. These false positives (two coincided with IAV co-detection) have been at least partially associated with software version 7.2.7 of the Panther Fusion^®^ platform or the crosstalk correction algorithm [45]. Although the manufacturer has recently implemented an adaptive crosstalk correction to mitigate inter-channel artifacts in high viral-load samples, this study was performed using software version 7.2.7 [46]. In the SC2-positive agreed specimen that showed variable detection across six replicates, APRA detected only the SC2 *S*-gene target; this pattern may reflect target-specific performance, as *RdRp* detection sensitivity for that assay has been reported to exceed *S*-gene sensitivity [43]. This observation supports the added robustness of multitarget detection strategies for high-mutation RNA respiratory viruses.

Co-infections involving IAV, IBV, SC2, and RSV have been previously described [47,48,49]. Although infrequent, their detection has prognostic value because it can support risk stratification for complications and mortality [50,51]. From an epidemiological standpoint, the identification of co-infections may also signal periods of intensified viral circulation and inform the need for more active surveillance [52]. In our study, the mixed-infection frequency (2.09%) falls within the post-pandemic range commonly reported in the literature (0.3–10%) [16,51,52,53,54,55]. In this study, the most frequent co-infection was SC2 + RSV, which is consistent with the temporal overlap of three outbreaks during the sample-selection period (Supplementary Figure S7). This distribution may explain why SC2 and RSV were the most frequently represented viruses among co-infection specimens (3 of 7), followed by IAV and IBV (2 of 7). Differences in co-infection detectability across the three RT-qPCR assays and the consensus reference standard are consistent with the false-negative and false-positive patterns discussed above. As recently described for a multiplex IAV typing assay [56], apparent false negatives in consensus-defined co-infected samples may reflect preferential amplification of the higher viral-load target, which can occur in multiplex settings and may suggest some degree of competitive effect among targets/oligos. Despite these differences, agreement for co-infection detection remained high across assays.

Finally, several limitations should be acknowledged. This was a single-center study, which may limit extrapolation to other regions and epidemiological contexts. Although samples were collected over an extended period, the overall cohort size was modest, and no demographic, epidemiological, or clinical data were available; therefore, potential associations with patient factors or outcomes could not be explored. Because specimens were de-identified residual samples stored retrospectively, the observed detection frequencies reflect only the analyzed cohort and should not be interpreted as population prevalence. Moreover, the analytical sensitivity estimates were generated using synthetic evaluation materials with target concentrations not traceable to a certified reference standard; thus, LoD values may not be directly comparable across studies and may not fully reflect performance in clinical matrices. Finally, discordant results were not adjudicated using an independent orthogonal method (e.g., sequencing or an additional reference assay), which limits definitive attribution of assay-specific errors. Despite these limitations, the study provides a technically relevant analytical and clinical performance assessment of the proposed quadruplex approach.

## 5. Conclusions

The quadruplex laboratory-developed respiratory assay (LDRA), based on the CDC Flu-SC2 chemistry and expanded with RSV detection, demonstrated strong analytical performance and diagnostic estimates comparable to those of commercial comparator assays. These findings support LDRA as a practical multiplex option for the detection of IAV, IBV, SC2, and RSV, with potential utility for diagnostic testing and respiratory virus surveillance. Implementation on the fully automated Panther Fusion^®^ platform may facilitate timely reporting and scalable workflows in medium- to high-throughput clinical laboratories.

## Figures and Tables

**Table 1:** Primers and probes of the quadruplex LDRA.

Assay_Oligo Type	Target Gene *	Nucleotide Position *	Oligo Sequence (5′→3′)	Tm ** (°C)	Amplicon Size (nt)	Ref.
IAV_fwd1	*M*	143–167	CAA GAC CAA TCY TGT CAC CTC TGA C	65.4 ^#^	106	[19]
IAV_fwd2		143–167	CAA GAC CAA TYC TGT CAC CTY TGA C	65 ^#^		
IAV_rev1		248–226	GCA TTY TGG ACA AAV CGT CTA CG	64.3 ^#^		
IAV_rev2		248–226	GCA TTT TGG ATA AAG CGT CTA CG	62.2		
IAV_prb ^1^		224–201	/FAM/TGC AGT CCT/ZEN/CGC TCA CTG GGC ACG/IABkFQ/	73.1		
IBV_fwd	*NS2*	758–779	TCC TCA AYT CAC TCT TCG AGC G	64.7 ^#^	103	[19]
IBV_rev		860–840	CGG TGC TCT TGA CCA AAT TGG	63.8		
IBV_prb ^2^		802–828	/YakYel/CCA ATT CGA/ZEN/GCA GCT GAA ACT GCG GTG/IABkFQ/	70.7		
SC2_fwd	*N*	29,463–29,485	CTG CAG ATT TGG ATG ATT TCT CC	61.7	92	[19]
SC2_rev		29,554–29,530	CCT TGT GTG GTC TGC ATG AGT TTA G	65.2		
SC2_prb ^3^		29,491–29,520	/TexRd-XN/ATT GCA ACA/TAO/ATC CAT GAG C**M**G TGC TGA CTC/IAbRQSp/	70.9 ^#^		
RSV A/B_fwd	*M*	3226–3249	GGC AAA TAT GGA AAC RTA CGT GAA	62.9 ^#^	80	This study
RSV A_rev		3308–3281	CTT TTT CTA RGA CAT TGT ATT GAA CAG C	62.5 ^#^		
RSV B_rev		3308–3281	CTT TTT CTA GAA CAT TGT AYT GRA CAG C	63 ^#^		
RSV A/B_prb ^4^		3278–3252	/CY5/CTG TGT ATG/TAO/TGG AGC CYT CGT GAA GYT/IAbRQSp/	69.2 ^#^		
RNaseP_fwd	*Human RNase P*	28–46	AGA TTT GGA CCT GCG AGC G	64.4	65	[19]
RNaseP_rev		92–73	GAG CGG CTG TCT CCA CAA GT	66.2		
RNaseP_prb ^5^		49–71	/CY5.5/TTC TGA CCT GAA GGC TCT GCG CG/IAbRQSp/	70		

* Nucleotide positions are indicated as location in the sequences for the Matrix (*M*) gene (Genbank accession number NC_026431.1) of influenza A virus (IAV), Nonstructural protein (*NS2*) gene (Genbank accession number NC_002211.1) of influenza B virus (IBV), Nucleocapsid (*N*) gene (Genbank accession number NC_045512.2) of SARS-CoV-2 (SC2), *M* gene (Genbank accession number NC_001803.1) of respiratory syncytial virus (RSV), or human ribonuclease P (*RNase P*) gen, subunit p30 (Genbank accession number NM_004613.4). ** Melting temperature (Tm) was estimated using OligoAnalyzer (Integrated DNA Technologies, Coralville, IA, USA) according to the [22] algorithm, assuming: concentration of oligos, 200 nM; concentration of divalent ions, 3 mM; concentration of monovalent ions, 50 mM; and concentration of deoxyribonucleoside triphosphate (dNTP), 0.8 mM. ^#^ Indicates average of the Tm for oligos containing degenerate nucleotides. ^1^ Probe labeled at the 5′-end with the reporter molecule 6-carboxyfluorescein (FAM), with a ZEN™ quencher between the 9th and 10th nucleotide, and with an Iowa Black FQ quencher (IABkFQ) at the 3′-end. ^2^ Probe labeled at the 5′-end with the Yakima Yellow (YakYel) reporter, with a ZEN™ quencher between the 9th and 10th nucleotide, and with an Iowa Black FQ quencher (IABkFQ) at the 3′-end. ^3^ Probe labeled at the 5′-end with the Texas Red-XN (TexRd-XN) reporter, with a TAO quencher between the 9th and 10th nucleotide, and with an Iowa Black RQ quencher (IAbRQSp) at the 3′-end. The modified nucleotide in the probe is highlighted in bold and underlined (M instead of A). ^4^ Probe labeled at the 5′-end with the Cyanine 5 (Cy5) reporter, with a TAO quencher between the 9th and 10th nucleotide, and with an Iowa Black RQ quencher (IAbRQSp) at the 3′-end. ^5^ Probe labeled at the 5′-end with the Cyanine 5.5 (Cy5.5) reporter between the 9th and 10th nucleotide, and with an Iowa Black RQ quencher (IAbRQSp) at the 3′-end. fwd, forward primer; rev, reverse primer; prb, probe; Ref., bibliographic references.

**Table 2:** Amplification efficiency analysis per target in a multiplex setting (LDRA).

Target	R^2^ *	Slope (95% CI)		*E* ** (95% CI)	
IAV	0.9998	−3.38	(−3.44–−3.33)	97%	(95.3–99.7%)
IBV	0.9995	−3.38	(−3.47–−3.29)	98%	(94.3–101.5%)
SC2	0.9978	−3.22	(−3.4–−3.05)	104%	(96.9–112.8%)
RSV A	0.9995	−3.20	(−3.28–−3.12)	105%	(101.7–109.3%)
RSV B	0.9966	−3.31	(−3.54–−3.09)	100%	(91.8–110.6%)

* Goodness of fit. ** Amplification efficiency. IAV, influenza A virus; IBV, influenza B virus; SC2, SARS-CoV-2; RSV A, respiratory syncytial virus type A; RSV B, respiratory syncytial virus type B.

**Table 3:** Limit of detection of the LDRA assay per target sequence/pathogen.

Pathogen	LoD (95% CI)			
	Copies/mL ^1^		Copies/Reaction	
IAV	2541	(1981–3899)	37.8	(29.5–58.0)
IBV	936	(736–1420)	13.9	(11.0–21.1)
SC2	643	(496–982)	9.6	(7.4–14.6)
RSV A	824	(648–1208)	12.3	(9.6–18.0)
RSV B	1690	(1374–2313)	25.1	(20.4–34.4)

^1^ Concentration of the target sequence in the primary sample (UTM), before mixing with SLM. LoD, limit of detection; IAV, influenza A virus; IBV, influenza B virus; SC2, SARS-CoV-2; RSV A, respiratory syncytial virus type A; RSV B, respiratory syncytial virus type B.

**Table 4:** Analytical specificity: cross-reactivity of the LDRA assay.

Pathogen	Variant, Subtype, Lineage or Strain	Source	Result			
			IAV	IBV	SC2	RSV
Adenovirus 1	n/a	NATRVP 2.1	−	−	−	−
Adenovirus 3	n/a		−	−	−	−
Adenovirus 31	n/a		−	−	−	−
*Bordetella parapertussis*	A747		−	−	−	−
*Bordetella pertussis*	A639		−	−	−	−
*Chlamydia pneumoniae*	CWL-029		−	−	−	−
Coronavirus 229E	n/a		−	−	−	−
Coronavirus HKU-1	n/a		−	−	−	−
Coronavirus NL63	n/a		−	−	−	−
Coronavirus OC43	n/a		−	−	−	−
Influenza A	A/NY/02/09 (H1N1pdm09)		+	−	−	−
Influenza B	B/Florida/02/06 (Victoria)		−	+	−	−
*Mycoplasma pneumoniae*	M129		−	−	−	−
Metapneumovirus 8	Peru6-2003		−	−	−	−
Parainfluenza 1	n/a		−	−	−	−
Parainfluenza 2	n/a		−	−	−	−
Parainfluenza 3	n/a		−	−	−	−
Parainfluenza 4	n/a		−	−	−	−
Rhinovirus 1A	n/a		−	−	−	−
RSV A	n/a		−	−	−	+
SARS-CoV-2	USA-WA1/2020		−	−	+	−
Adenovirus 14	n/a	RTX1-5QC	−	−	−	−
Enterovirus A16	n/a		−	−	−	−
Enterovirus 68	n/a		−	−	−	−
Metapneumovirus A2	n/a		−	−	−	−
Rhinovirus 16	n/a		−	−	−	−
Adenovirus A	n/a	IDR-C 2024	−	−	−	−
Enterovirus A71	n/a	IDR-A 2023	−	−	−	−
*Legionella pneumophila*	Philadelphia Group 1		−	−	−	−
Metapneumovirus B2	n/a		−	−	−	−

n/a, information not provided by the manufacturer; NATRVP 2.1, NATtrol Respiratory Verification Panel 2.1; RTX1-5QC, Respiratory Multiplex (1 to 5) Q Control; EQA, external quality assessment; CAP, College of American Pathologists; IDR, CAP Infectious Disease, Respiratory EQA Programme.

**Table 5:** Analytical specificity: in vitro inclusivity of the LDRA assay.

Pathogen	Variant, Subtype, Lineage or Strain	Source	Result			
			IAV	IBV	SC2	RSV
Influenza A	A/NY/02/09 (H1N1pdm09)	NATRVP 2.1	+	−	−	−
	A/NewCaledonia/20/99 (H1N1sea)		+	−	−	−
	A/Brisbane/10/07 (H3N2)		+	−	−	−
	A/Brisbane/02/2018 (H1N1pdm09)	IDR-A 2023	+	−	−	−
	A/HongKong/2671/2019 (H3N2)	IDR-B 2023	+	−	−	−
	A/Victoria/2570/2019 (H1N1pdm09)	ID3-C 2024	+	−	−	−
	A/Cambodia/e0826360/2020 (H3N2)	ID3-A 2024	+	−	−	−
	A/Kansas/14/2017 (H3N2)	ID3-B 2023	+	−	−	−
	A/Netherlands/1250/2016 (H1N1pdm09)	INFTP24	+	−	−	−
	A/Hong Kong/213/2003 (H5N1)		+	−	−	−
	A/Netherlands/398/2014 (H3N2)		+	−	−	−
	A/Netherlands/2393/2015 (H3N2)		+	−	−	−
	A/Mallard/Netherlands/2/2009 (H7N7)		+	−	−	−
	A/NIBRG-14 (H5N1) *	ASRC	+	−	−	−
	A/Brisbane/59/2007 (H1N1sea)		+	−	−	−
	A/Perth/16/2009 (H3N2)		+	−	−	−
	A/cattle/Texas/56283/2024 (H5N1)	APLH5N1	+	−	−	−
Influenza B	B/Florida/02/06 (Victoria)	NATRVP 2.1	−	+	−	−
	B/Washington/02/2019 (Victoria)	ID3-A 2024	−	+	−	−
	B/Brisbane/60/2008 (Yamagata)	ATSFR	−	+	−	−
	B/Phuket/3073/2013 (Yamagata)	ID3-B 2024	−	+	−	−
SARS-CoV-2	B.1.1.7 (Alpha)	ASRC	−	−	+	−
	B.1.351 (Beta)		−	−	+	−
	P.1 (Gamma)		−	−	+	−
	B.1.617.2 (Delta)		−	−	+	−
	BA.1 (Omicron)		−	−	+	−
	A (USA-WA1/2020)	ID3-A 2024	−	−	+	−
	B (Italy-INMI1/2020)	ID3-B 2024	−	−	+	−
RSV	Type A	NATRVP 2.1	−	−	−	+
		RTX1QC	−	−	−	+
		ID3-A 2024	−	−	−	+
	Type B	RTX5QC	−	−	−	+
		IDR-B 2024	−	−	−	+
	4/2015 (Type B)	IDR-B 2023	−	−	−	+
	9320 (Type B)	ATSFR	−	−	−	+

* A/reassortant/NIBRG-14 (Viet Nam/1194/2004 × Puerto Rico/8/1934). H1N1sea, seasonal H1N1; H1N1pdm09, H1N1 derived from the 2009 pandemic; NATRVP 2.1, NATtrol Respiratory Verification Panel 2.1; EQA, external quality assessment; CAP, College of American Pathologists; QCMD, Quality Control for Molecular Diagnostics; INFTP24, QCMD 2024 Influenza Typing EQA Programme; APLH5N1, AccuPlex™ H5N1 Influenza Reference Material Kit; IDR, CAP Infectious Disease, Respiratory EQA Programme; ID3, CAP Nucleic Acid Amp, Respiratory Ltd. EQA Programme; ATSFR, Amplirun^®^ Total SARS-CoV-2/FluA/FluB/RSV Control (Swab); ASRC, Amplirun^®^ SARS-CoV-2 RNA Controls; RTX#QC, Respiratory Multiplex (1 to 5) Q Control.

**Table 6:** LDRA assay variability.

Member Panel	Content	Pathogen	Level	Agreed/*n*	Agreement	Mean Ct	Within Lot		Between Lot		Between Instrument		Between Run/Day		Within Run/Day		Within Laboratory (Total)	
							SD (95% CI)	CV	SD (95% CI)	CV	SD (95% CI)	CV	SD (95% CI)	CV	SD (95% CI)	CV	SD (95% CI)	CV
1	Neg	n/a	n/a	112/112	100%	n/a	n/a	n/a	n/a	n/a	n/a	n/a	n/a	n/a	n/a	n/a	n/a	n/a
2	IAV/SC2	IAV	Mod	112/112	100%	27.93	0.16	0.6%	0.13	0.5%	0	0%	0.26	0.9%	0.21	0.7%	0.33	1.2%
							(0.14–0.2)		(0.07–0.21)		(0–0.12)		(0.18–0.43)		(0.19–0.25)		(0.28–0.48)	
		SC2	Low	112/112	100%	34.40	0.32	0.9%	0	0%	0.4	1.2%	0	0%	0.51	1.15%	0.51	1.5%
							(0.27–0.4)		(0–0.21)		(0.27–0.66)		(0–0.33)		(0.42–0.74)		(0.46–0.66)	
3	SC2/IAV	IAV	Low	111/112 ^1^	99.1%	34.84	0.95	2.7%	0	0%	0.3	0.8%	0	0%	1	2.9%	1	2.9%
							(0.8–1.17)		(0–0.55)		(0–0.68)		(0–0.43)		(0.9–1.21)		(0.91–1.17)	
		SC2	Mod	112/112	100%	25.49	0.12	0.5%	0.1	0.4%	0.43	1.7%	0	0%	0.46	1.8%	0.46	1.8%
							(0.1–0.14)		(0.06–0.16)		(0.31–0.69)		(0–0.22)		(0.35–0.7)		(0.4–0.59)	
4	IBV/RSV	IBV	Mod	112/112	100%	25.30	0.15	0.6%	0.1	0.4%	0.14	0.5%	0	0%	0.23	0.9%	0.23	0.9%
							(0.13–0.19)		(0.03–0.17)		(0.05–0.25)		(0–0.12)		(0.2–0.32)		(0.21–0.29)	
		RSV	Low	111/112 ^1^	99.1%	34.72	0.36	1%	0.17	0.5%	0.34	1%	0	0%	0.52	1.5%	0.52	1.5%
							(0.3–0.44)		(0–0.33)		(0.18–0.6)		(0–0.24)		(0.44–0.72)		(0.48–0.64)	
5	RSV/IBV	IBV	Low	111/112 ^2^	99.1%	32.21	0.31	1%	0.21	0.7%	0	0%	0.19	0.6%	0.38	1.2%	0.42	1.3%
							(0.26–0.38)		(0.05–0.35)		(0–0.22)		(0.06–0.34)		(0.34–0.45)		(0.38–0.52)	
		RSV	Mod	111/112 ^2^	99.1%	26.07	0.37	1.4%	0.12	0.5%	0.15	0.6%	0	0%	0.42	1.6%	0.42	1.6%
							(031–0.46)		(0–0.29)		(0–0.34)		(0–0.2)		(0.37–0.53)		(0.38–0.5)	

^1^ Outlier. ^2^ Invalid result by system error. *n*, total number of replicates; SD, standard deviation; CV, coefficient of variation; 95% CI, 95% confidence interval; Neg, negative; IAV, influenza A virus; IBV, influenza B virus; SC2, SARS-CoV-2; RSV, respiratory syncytial virus; Mod, moderate level.

**Table 7:** Method agreement.

Comparator RT-qPCR Assay	Target		Candidate RT-qPCR Assay: LDRA		*n*	NPA (95% CI)	PPA (95% CI)	OPA (95% CI)	*k* (95% CI)
			+	−					
PFRA	IAV	+	65	0	405	100%	100%	100%	1.000
		−	0	340		(98.9–100%)	(94.4–100%)	(99.1–100%)	(1.000–1.000)
	IBV	+	79	0	405	100%	100%	100%	1.000
		−	0	326		(98.8–100%)	(95.4–100%)	(99.1–100%)	(1.000–1.000)
	SC2	+	88 *	4	405	100%	95.7%	99.0%	0.971
		−	0	313		(98.8–100%)	(89.3–98.3%)	(97.5–99.6%)	(0.944–0.999)
	RSV	+	111	1	405	100%	99.1%	99.8%	0.994
		−	0	293		(98.7–100%)	(95.1–99.8%)	(98.6–100%)	(0.982–1.000)
APRA	IAV	+	64	0	405	99.7%	100%	99.8%	0.991
		−	1	340		(98.4–99.9%)	(94.3–100%)	(98.6–100%)	(0.973–1.000)
	IBV	+	78	0	405	99.7%	100%	99.8%	0.992
		−	1	326		(98.3–99.9%)	(95.3–100%)	(98.6–100%)	(0.977–1.000)
	SC2	+	88 *	0	405	100%	100%	100%	1.000
		−	0	317		(98.8–100%)	(95.8–100%)	(99.1–100%)	(1.000–1.000)
	RSV	+	110	0	405	99.7%	100%	99.8%	0.994
		−	1	294		(98.1–99.9%)	(96.6–100%)	(98.6–100%)	(0.982–1.000)

* One of the samples was considered consistently positive for SC2, even though differences in the frequency of hit rates were found when analyzing six replicates of the same sample: 2 out of 6 using LDRA, 3 out of 6 using PFRA, and 6 out of 6 using APRA (positivity only for the SC2 *S* gene). LDRA, laboratory-developed respiratory assay (CDC Flu-SC2 + LDT RSV); PFRA, Panther Fusion^®^ SARS-CoV-2/Flu A/B/RSV assay; APRA, Allplex™ SARS-CoV-2/FluA/FluB/RSV assay; IAV, influenza A virus; IBV, influenza B virus; SC2, SARS-CoV-2; RSV, respiratory syncytial virus; *n*, total number of samples; NPA, negative percent agreement; PPA, positive percent agreement; OPA, overall percent agreement; *κ*, *κ*-index; 95% CI, 95% confidence interval.

**Table 8:** Diagnostic performance parameters.

RT-qPCR Assay	Target	TP	FP	FN	TN	*n*	PR (95% CI)	dSens (95% CI)	dSpec (95% CI)	PPV (95% CI)	NPV (95% CI)
LDRA	IAV	65	0	0	340	405	16.0%	100%	100%	100%	100%
							(12.8–19.9%)	(94.4–100%)	(98.9–100%)	(94.5–100%)	(98.9–100%)
	IBV	79	0	0	326	405	19.5%	100%	100%	100%	100%
							(15.9–23.6%)	(95.4–100%)	(98.8–100%)	(95.4–100%)	(98.9–100%)
	SC2	88	0	0	317	405	21.7%	100%	100%	100%	100%
							(18.0–26.0%)	(95.8–100%)	(98.8–100%)	(95.9–100%)	(98.8–100%)
	RSV	111	0	0	294	405	27.4%	100%	100%	100%	100%
							(23.3–32.0%)	(96.7–100%)	(98.7–100%)	(96.7–100%)	(98.8–100%)
PFRA	IAV	65	0	0	340	405	16.0%	100%	100%	100%	100%
							(12.8–19.9%)	(94.4–100%)	(98.9–100%)	(94.5–100%)	(98.9–100%)
	IBV	79	0	0	326	405	19.5%	100%	100%	100%	100%
							(15.9–23.6%)	(95.4–100%)	(98.8–100%)	(95.4–100%)	(98.9–100%)
	SC2	88	4	0	313	405	22.7%	100%	98.7%	95.7%	100%
							(18.9–27.0%)	(95.8–100%)	(96.8–99.5%)	(89.3–98.3%)	(98.8–100%)
	RSV	111	1	0	293	405	27.7%	100%	99.7%	99.1%	100%
							(23.5–32.2%)	(96.7–100%)	(98.1–99.9%)	(94.0–99.9%)	(98.8–99.9%)
APRA	IAV	64	0	1	340	405	15.8%	98.5%	100%	100%	99.7%
							(12.6–19.7%)	(91.8–99.7%)	(98.9–100%)	(94.4–100%)	(98.0–100%)
	IBV	78	0	1	326	405	19.3%	98.7%	100%	100%	99.7%
							(15.7–23.4%)	(93.2–99.8%)	(98.8–100%)	(95.4–100%)	(97.9–100%)
	SC2	88	0	0	317	405	21.7%	100%	100%	100%	100%
							(18.0–26.0%)	(95.8–100%)	(98.8–100%)	(95.9–100%)	(98.8–100%)
	RSV	110	0	1	294	405	27.2%	99.1%	100%	100%	99.7%
							(23.1–31.7%)	(95.1–99.8%)	(98.7–100%)	(96.7–100%)	(97.7–100%)

LDRA, laboratory-developed respiratory assay (CDC Flu-SC2 + LDT RSV); PFRA, Panther Fusion^®^ SARS-CoV-2/Flu A/B/RSV assay; APRA, Allplex™ SARS-CoV-2/FluA/FluB/RSV assay; IAV, influenza A virus; IBV, influenza B virus; SC2, SARS-CoV-2; RSV, respiratory syncytial virus; TP, true positive; FP, false positive; FN, false negative; TN, true negative; *n*, total number of samples; PR, positive rate; dSens, diagnostic sensitivity; dSpec, diagnostic specificity; PPV, positive predictive value; NPV, negative predictive value; 95% CI, 95% confidence interval.

**Table 9:** RT-qPCR assay capability comparison for detecting mixed infections.

RT-qPCR Assay		Consensus		*n*	SinPA (95% CI)	MixPA (95% CI)	OPA (95% CI)	*κ* (95% CI)
		Mix	Sin					
LDRA	Mix	7	0	335	100%	100%	100%	1.000
	Sin	0	328		(98.8–100%)	(64.6–100%)	(98.9–100%)	(1.000–1.000)
PFRA	Mix	7	3	335	99.1%	100%	99.1%	0.819
	Sin	0	325		(97.3–99.7%)	(64.6–100%)	(97.4–99.7%)	(0.619–1.000)
APRA	Mix	5	0	335	100%	71.4%	99.4%	0.830
	Sin	2	328		(98.8–100%)	(35.9–91.8%)	(97.8–99.8%)	(0.599–1.000)

LDRA, laboratory-developed respiratory assay (CDC Flu-SC2 + LDT RSV); PFRA, Panther Fusion^®^ SARS-CoV-2/Flu A/B/RSV assay; APRA, Allplex™ SARS-CoV-2/FluA/FluB/RSV assay; *n*, total number of positive samples; Mix, mixed infection detected; Sin, single infection detected; SinPA, single-infection percent agreement; MixPA, mixed-infection percent agreement; OPA, overall percent agreement; *κ*, *κ*-index; 95% CI, 95% confidence interval.

## Data Availability

The original contributions presented in this study are included in the article/Supplementary Materials. Further inquiries can be directed to the corresponding author.

## Supplementary Materials

The following supporting information can be downloaded at: https://www.mdpi.com/article/10.3390/microorganisms14010167/s1, Supplementary Figures and Tables.docx (Figure S1: Variability and coverage plot: conserved region of the M gene of RSV; Figure S2: Variability and coverage plot: conserved region of the L gene of RSV; Figure S3: Comparative representation of three sets of oligos for RSV detection; Figure S4: Comparative inclusivity; Figure S5: Multiplex compatibility analysis; Figure S6: Probit regression; Figure S7: Positivity (relative frequency) of the influenza A virus (IAV), influenza B virus (IBV), respiratory syncytial virus (RSV) and SARS-CoV-2 (SC2) infections between 1 October 2023 and 30 September 2025; Table S1: Microorganisms whose sequences were employed for the in silico cross-reactivity evaluation of the primers and probe used in the LDT RSV assay; Table S2: Sequence of the Ultramer–duplex synthetic controls; Table S3: Optimized PPR for the quadruplex LDRA; Table S4: Inclusivity analysis of the LDT RSV assay; Table S5: Hit rate by dilution level for the analytical sensitivity evaluation panel of the quadruplex LDRA assay; Table S6: Inclusivity analysis of the CDC Flu-SC2 detection assay); File S1: Conserved Region Search_WO Redundancies.txt; File S2: Positional Nt Numerical Summary_WO Redundancies.txt; File S3: Entropy & Coverage_WO Redundancies_M gene.xls; File S4: Entropy & Coverage_WO Redundancies_L gene.xls; File S5: LDT_Flu-SC2-RSV_v2 (Open Access v3.0).pdf; File S6: In silico homology analysis (BLAST).txt; File S7: In silico cross-reactivity (BLAST).txt; File S8: hDNA cross-reactivity (MFEprimer3.1).txt; File S9: Genbank_RSV A_Inclusivity & Clustering.xls; File S10: Genbank_RSV B_Inclusivity & Clustering.xls; File S11: GISAID_RSV A_Inclusivity & Clustering.xls; File S12: GISAID_RSV B_Inclusivity & Clustering.xls. The supplementary materials cited Refs. [21,29,40,57,58].

## Abbreviations

The following abbreviations are used in this manuscript:

95% CI	95% confidence interval
ANOVA	Analysis of variance
APRA	Allplex™ SARS-CoV-2/FluA/FluB/RSV assay (Allplex respiratory assay)
ASRC	Amplirun^®^ SARS-CoV-2 RNA Controls
CAP	College of American Pathologists
CDC	Centers for Disease Control and Prevention
CV	Coefficient of variation
Cy5	Cyanine 5 dye
Cy5.5	Cyanine 5.5 dye
DNA	Deoxyribonucleic acid
dSens	Diagnostic sensitivity
dSpec	Diagnostic specificity
*E*	Amplification efficiency
EQA	External quality assessment
*E*-value	Expectation value
FAM	6-carboxifluoresceína dye
FN	False negative
FP	False positive
fwd	Forward primer
GISAID	Global Initiative on Sharing Influenza Data
H1N1pdm09	H1N1 subtype derived from the 2009 pandemic
H1N1sea	Seasonal H1N1 subtype
IAV	Influenza A virus
IBV	Influenza B virus
IC	Internal control
IC-S	Panther Fusion^®^ Internal Control-S
ID3	CAP Nucleic Acid Amp, Respiratory Ltd. EQA Programme
IDR	CAP Infectious Disease, Respiratory EQA Programme
INFTP24	QCMD 2024 Influenza Typing EQA Programme
*κ*	*Kappa* index
LDRA	Laboratory-developed respiratory assay
LDT	Laboratory-developed test
LDT RSV	RSV A/B RT-qPCR assay
LoD	Limit of detection
MAFFT	Multiple alignment using fast Fourier transform
MixPA	Mixed-infection percent agreement
MMX	Master mix
*n*	Total number of samples/replicates
NAAT	Nucleic acid amplification test
NATRVP 2.1	NATtrol Respiratory Verification Panel 2.1
NCBI	National Center for Biotechnology Information
Neg	Negative result
NPA	Negative percent agreement
NPV	Negative predictive value
OPA	Overall percent agreement
PFRA	Panther Fusion^®^ SARS-CoV-2/Flu A/B/RSV assay (Panther Fusion respiratory assay)
PPA	Positive percent agreement
PPR	Primers and probes reconstitution solution
PPV	Positive predictive value
PR	Positive rate
prb	Probe
QCMD	Quality Control for Molecular Diagnostics
qPCR	Real-time PCR or Quantitative PCR
RdRp	RNA-dependent RNA polymerase
rev	Reverse primer
RFU	Relative fluorescence units
RNA	Ribonucleic acid
RNase P	Human RNase P
RSV	Respiratory syncytial virus
RT	Reverse transcription
RT-qPCR	Real-time PCR with reverse transcription
RTX#QC	Respiratory Multiplex (1 to 5) Q Control
SC2	SARS-CoV-2
SD	Standard deviation
SinPA	Single-infection percent agreement
SLM	Medium of the Panther Fusion^®^ Specimen Lysis Tube (specimen lysis medium)
SOP	Standard operating procedure
TexRd-XN	Texas Red-XN dye
Tm	Melting temperature
TN	True negative
TP	True positive
UTM	Universal transport medium
UTM/SLM	UTM from negative nasopharyngeal swabs in the SLM at the manufacturer-recommended proportion: 0.5 mL UTM per 0.71 mL SLM
WHO	World Health Organization
YakYel	Yakima Yellow dye
