# Supplementary material for: From Triplex to Quadruplex: Enhancing CDC’s Respiratory qPCR Assay with RSV Detection on Panther Fusion® Open Access™
Source: Microorganisms. 2026 Jan 12;14(1):167. doi: 10.3390/microorganisms14010167 (PMC12844171; doi:10.3390/microorganisms14010167)
Supplement: Supplementary file 1 [file microorganisms-14-00167-s001.zip › File S5. LDT_Flu-SC2-RSV_v2 (Open Access v3.0).pdf]

Protocol Definition: LDT-Flu/RSV/SC2 (Version 2.0.8.0)

Protocol Identification

Protocol Name	LDT-Flu/RSV/SC2
Version	2.0.8.0
Extraction Type	Low - Viral (2.10)
Results Processing	Qualitative
Author	Andy Caballero Mendez
Created On	10/20/2025 11:08:37 AM
Modified On	--
Exported On	10/20/2025 11:10:06 AM
Description	Detection and diferentiation of Influenza A virus (IAV), influenza B virus (IBV), SARS-CoV-2 and Respiratory Syncytial virus (RSV A/B) from respiratory samples.

Extraction & PCR Details

Sample Tube Cap Type	Hologic Pierceable Cap
Sample Aspiration Height	Low
Template	RNA or DNA
Template Volume	5 µL
Extraction Volume	360 µL
Reagent Kit	FCR-S/FER-S
Elution Buffer Volume	50 µL
Enzyme Cartridge Type	RNA/DNA Enzyme

Targets Setup

Selected	Channel	Example Dye	Excitation/Detection Wavelength (nm)	Enable Internal Control	Analyte Name	Additional Information (Optional)
Yes	1	FAM	460/517	No	FluA	Influenza A virus
Yes	2	HEX	532/563	No	FluB	Influenza B virus
Yes	3	ROX	586/625	No	SC2	SARS-CoV-2
Yes	4	Quasar 670	625/660	No	RSV	Respiratory Syncytial virus (RSV A/B)
Yes	5	Quasar 705	690/720	Yes	IC	Human RNase P

Protocol Definition: LDT-Flu/RSV/SC2 (Version 2.0.8.0)

Thermocycler Setup

Profile: FluA/B/RSV/SC2
Projected Thermocycler Runtime (hh:mm:ss): 00:54:17

Stage Name 1: Holding Stage
Number Of Cycles 1

Step Name Step 1
Temperature (°C) 46
Duration (mm:ss) 08:00
Optics On No

Step Name Step 2
Temperature (°C) 95
Duration (mm:ss) 02:00
Optics On No

Stage Name 2: Cycling Stage
Number Of Cycles 45

Step Name Step 1
Temperature (°C) 95
Duration (mm:ss) 00:05
Optics On No

Step Name Step 2
Temperature (°C) 58
Duration (mm:ss) 00:21
Optics On Yes

Parameters

Curve Correction Method Option 2
Enhanced Resolution Enabled No

Curve Correction

Analyte Name	Channel	Analysis Start Cycle	Baseline Correction	
			Enable	Slope Limit
FluA	1-FAM	10	Yes	50
FluB	2-HEX	10	Yes	50
SC2	3-ROX	10	Yes	50
RSV	4-Quasar 670	10	Yes	50
IC	5-Quasar 705	10	Yes	50

Protocol Definition: LDT-Flu/RSV/SC2 (Version 2.0.8.0)

Crosstalk Correction

Analyte Name	Emit\Receive	1	2	3	4	5
FluA	1-FAM	--	1.00 %	0.00 %	0.00 %	0.00 %
FluB	2-HEX	5.00 %	--	1.00 %	0.50 %	0.00 %
SC2	3-ROX	0.00 %	2.00 %	--	2.80 %	0.00 %
RSV	4-Quasar 670	0.00 %	0.00 %	2.00 %	--	1.00 %
IC	5-Quasar 705	0.00 %	0.00 %	0.00 %	2.00 %	--

Positivity Criteria

Analyte Name	Channel	Ct Threshold	Minimum Slope at Threshold		Maximum Ct	
			Enable	Value	Enable	Value
FluA	1-FAM	200	No	--	No	--
FluB	2-HEX	200	No	--	No	--
SC2	3-ROX	100	No	--	No	--
RSV	4-Quasar 670	50	No	--	No	--
IC	5-Quasar 705	100	No	--	Yes	38

Channel Validity Criteria

Analyte Name	Channel	Minimum Background Fluorescence		Maximum Background Fluorescence		Lowest Valid Ct	
		Enable	Value	Enable	Value	Enable	Value
FluA	1-FAM	No	--	No	--	Yes	12
FluB	2-HEX	No	--	No	--	Yes	12
SC2	3-ROX	No	--	No	--	Yes	12
RSV	4-Quasar 670	No	--	No	--	Yes	12
IC	5-Quasar 705	No	--	No	--	No	--

Sample Validity Criteria

Internal Control (IC) in Channel 5-Quasar 705

Validity Criteria Make IC valid if any other channel is positive

Protocol Definition: LDT-Flu/RSV/SC2 (Version 2.0.8.0)

Controls

Control Failure Handling Invalidate Specimens
Control Set Expiration 1 days

Control Name	Control Type	Analyte Name	Channel	Minimum Ct Value	Maximum Ct Value
FluA/B/SC2/RSV_NC	Negative	IC	5-Quasar 705	20	38
FluA/B/SC2/RSV_PC	Positive	FluA	1-FAM	26	33
		FluB	2-HEX	26	33
		SC2	3-ROX	29	36
		RSV	4-Quasar 670	26	33

Reflex From Other Assay(s)

--

Reflex To Other Assay(s)

Result	Assay
FluA POS	LDT-FluA Typing

Export Settings

Protocol Lock Status On
Sample Results to LIS Mode Always send
Revision Comments The acceptability criteria for the positive control were adjusted.

Notes

--
