# Supplementary material for: From Triplex to Quadruplex: Enhancing CDC’s Respiratory qPCR Assay with RSV Detection on Panther Fusion® Open Access™
Source: Microorganisms. 2026 Jan 12;14(1):167. doi: 10.3390/microorganisms14010167 (PMC12844171; doi:10.3390/microorganisms14010167)
Supplement: Supplementary file 1 [file microorganisms-14-00167-s001.zip › Supplementary Figures and Tables.pdf]

Article

From Triplex to Quadruplex: Enhancing CDC's Respiratory qPCR Assay with RSV Detection on Panther Fusion® Open Access™

Supplementary Figures and Tables

1. Target sequence selection

The entropy calculation, as previously described [1], yielded two regions of minimal variability: a sequence of 139 bp between nucleotides 3222 and 3360 of the RSV *M* gene and a sequence of 110 bp between nucleotides 9056 and 9165 of the RSV *L* (polymerase) gene, considering the GenBank reference sequence with accession number NC_001803.1 (Figures S1 and S2).

For primer and probe design for RSV amplification and detection, the conserved sequence of the RSV *M* gene was selected because it is larger (139 bp vs. 110 bp) and contains a higher proportion of GC pairs (%GC: 42.45% vs. 28.18%). The larger the size of the conserved sequence, the greater the possibility of selecting functional oligosets. The higher the GC%, the greater the stability of the oligos, which translates into greater amplification efficiency.

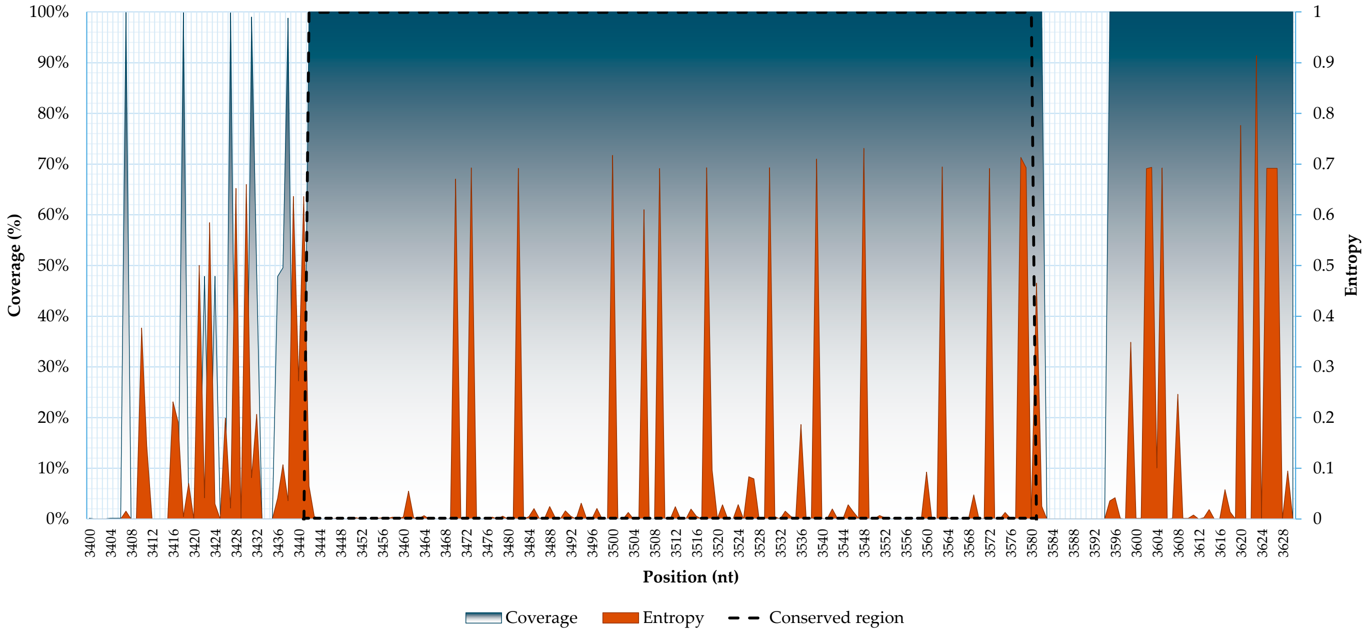

Figure S1. Variability and coverage plot: conserved region of the M gene of RSV. Coverage (blue-gray gradient) and entropy (brown) are shown vs. nucleotide positions (3,400–3,630 nt) of an alignment of 8,100 RSV A/B sequences retrieved from the NCBI Virus database (https://www.ncbi.nlm.nih.gov/labs/virus/, accessed on September 2025). The region of maximal coverage and minimal entropy (selected conserved region) is depicted between black dashed lines.

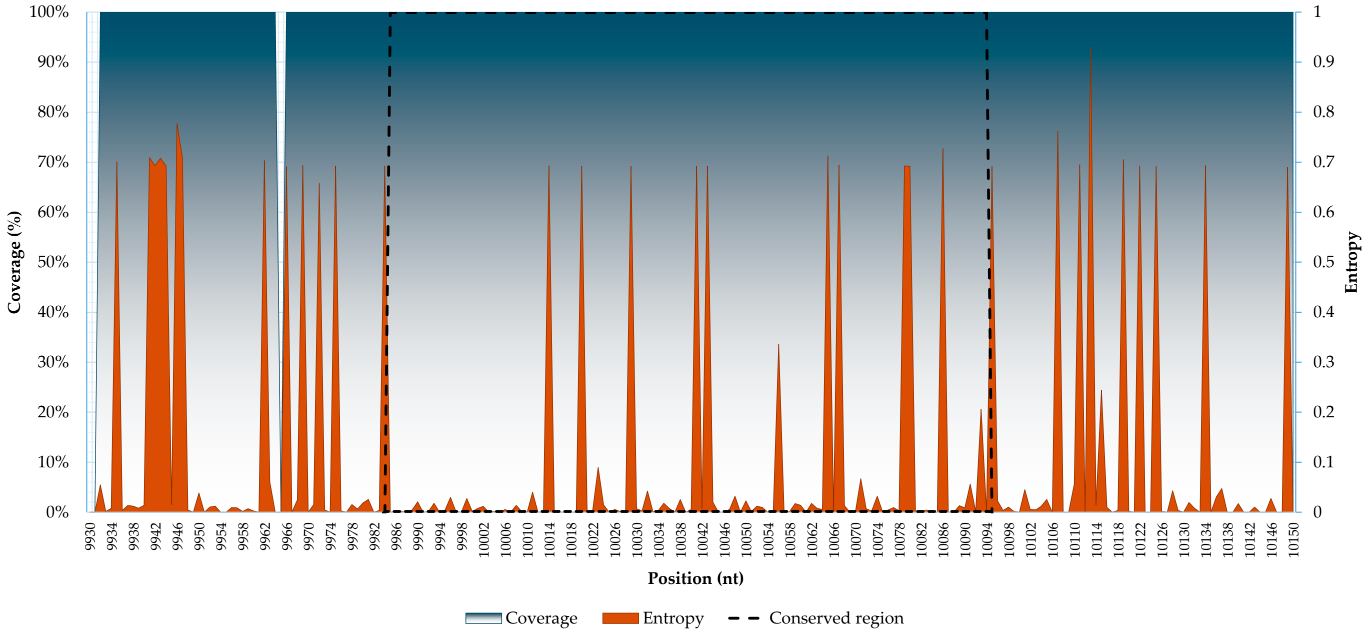

Figure S2. Variability and coverage plot: conserved region of the L gene of RSV. Coverage (blue-gray gradient) and entropy (brown) are shown vs. nucleotide positions (9,930–10,150 nt) of an alignment of 8,100 RSV (A/B) sequences retrieved from the NCBI Virus database (https://www.ncbi.nlm.nih.gov/labs/virus/, accessed on September 2025). The region of maximal coverage and minimal entropy is depicted between black dashed lines.

2. In silico *cross-reactivity*

The LDT RSV assay's oligo sequences exhibited low homology with non-RSV sequences. Low homology, and consequently a low probability of cross-reactivity, was defined as an *E*-value > 10^{-2}, regardless of the coverage or percentage of identity values (Table S1).

Table S1. Microorganisms whose sequences were employed for the *in silico* cross-reactivity evaluation of the primers and probe used in the LDT RSV assay.

Microorganism	Taxid	Microorganism (Cont'd)	Taxid (Cont'd)
Acinetobacter baumannii complex	909768	Human rhinovirus sp.	169066
Actinobacteria	1760	Influenza B virus	11520
Bordetella parapertussis	519	Influenza C virus	11552
Bordetella pertussis	520	Influenza A virus	197911
Candida	1535326	*Klebsiella aerogenes*	548
Chlamydophila pneumoniae	83558	*Klebsiella oxytoca*	571
Corynebacterium diphtheriae	1717	*Klebsiella pneumoniae*	573
Dolosigranulum	29393	*Lactobacillus plantarum*	1590
Eikenella sp.	540	*Legionella pneumophila*	446
Enterobacter cloacae complex	354276	Measles morbillivirus	11234
Epstein-Barr virus	10376	MERS-CoV	1335626
Escherichia coli	562	*Moraxella catarrhalis*	480
Firmicutes	1239	Mumps orthorubulavirus	2560602
Fusobacterium sp.	68766	*Mycobacterium tuberculosis*	1773
Haemophilus influenzae	727	*Mycoplasma pneumoniae*	2104
HCoV-229E	11137	*Neisseria*	482
HCoV-HKU1	290028	*Neisseria meningitidis*	487
HCoV-NL63	277944	*Neisseria mucosa*	488

HCoV-OC43	31631	*Pneumocystis jirovecii*	42068
SARSr-CoV	694009	*Prevotella* sp.	59823
Hepatitis B virus	10407	*Propionibacterium* sp.	1977903
Hepatitis C virus	11103	*Proteobacteria*	1224
Herpes simplex virus 1	10298	*Proteus* sp	229037
Herpes simplex virus 2	10310	*Pseudomonas aeruginosa*	287
HIV	11676	SARS-CoV-2	2697049
Human parainfluenza virus 1	12730	*Serratia marcescens*	615
Human parainfluenza virus 2	2560525	*Staphylococcus aureus*	1280
Human parainfluenza virus 3	11216	*Staphylococcus epidermidis*	1282
Human parainfluenza virus 4	2560526	*Streptococcus agalactiae*	1311
Human adenovirus sp.	1907210	*Streptococcus pneumoniae*	1313
Human cytomegalovirus HCMV	10359	*Streptococcus pyogenes*	1314
Human enterovirus	1193974	*Streptococcus salivarius*	1304
Human metapneumovirus	162145	*Streptococcus viridans*	78535
Human papillomavirus	10566	Varicella-zoster virus	10335

3. Synthetic controls: Ultramer™ duplex

The Ultramer™ duplex controls were synthesized by Integrated DNA Technologies (IDT, IA, USA) and purified by standard desalting. They were shipped lyophilized at 4 nmol (Supplementary Table S2).

As a positive control, a mixture of all synthetic controls was used in Specimen Transport Medium (STM), cat. PRD-04423 (Hologic, CA, USA): 80 µL of level 1.41×10^8 copies/mL of each synthetic control in an 80 mL STM bottle. As a negative control, a diluted preparation of the synthetic human RNase P control, Hs_RPP30 Positive Control, cat. 10006626 (IDT, IA, USA), was used: 80 µL (2×10^8 copies/mL) in an 80 mL STM bottle.

Table S2. Sequence of the Ultramer™ duplex synthetic controls.

Pathogen	Target gene	RefSeq [1]	Nucleotide position	Ultramer™ duplex sequence (5′→3′)	Length (bp)
IAV	*M*	NC_026431.1	87–286	AAG TGT CTT TGC AGG AAA GAA CAC AGA TCT TGA GGC TCT CAT GGA ATG GCT AAA GAC AAG ACC AAT CTT GTC ACC TCT GAC TAA GGG AAT TTT AGG ATT TGT GTT CAC GCT CAC CGT GCC CAG TGA GCG AGG ACT GCA GCG TAG ACG CTT TGT CCA AAA TGC CCT AAA TGG GAA TGG GGA CCC GAA CAA CAT GGA TAG AG	200
IBV	*NS2*	NC_002211.1	718–917	GAT GAT CGG ACA GTG GAG GAT GAA AAA GAT GGC CAT CGG ATC CTC AAC TCA CTC TTC GAG CGT TTT GAT GAA GGA CAT TCA AAG CCA ATT CGA GCA GCT GAA ACT GCG GTG GGA GTC TTA TCC CAA TTT GGT CAA GAG CAC CGA TTA TCA CCA GAA GAG GGA GAC AAT TAG ACT GGC CAC GGA AGA ACT TTA TCT CTT GA	200
SC2	*N*	NC_045512.2	29409–29608	CTC AAG CCT TAC CGC AGA GAC AGA AGA AAC AGC AAA CTG TGA CTC TTC TTC CTG CTG CAG ATT TGG ATG ATT TCT CCA AAC AAT TGC AAC AAT CCA TGA GCA GTG CTG ACT CAA CTC AGG CCT AAA CTC ATG CAG ACC ACA CAA GGC AGA TGG GCT ATA TAA ACG TTT TCG CTT TTC CGT TTA CGA TAT ATA GTC TAC TC	200
RSV A	*M*	NC_038235.1	3209–3408	CCC GGA AAA AAT CTA TAA TAT AGT TAC AAA AAA AGG AAA GGG TGG GGC AAA TAT GGA AAC ATA CGT GAA CAA GCT TCA CGA AGG CTC CAC ATA CAC AGC TGC TGT TCA ATA CAA TGT CTT AGA AAA AGA CGA TGA CCC TGC ATC ACT TAC AAT ATG GGT GCC CAT GTT CCA ATC ATC TAT GCC AGC AGA TTT ACT TAT AA	200
RSV B	*M*	NC_001781.1	3211-3410	ACC TGA CGA AAT TAA CAA TAT AGT AAC AAA AAA AGA ACA AGA TGG GGC AAA TAT GGA AAC ATA CGT GAA CAA GCT TCA CGA AGG CTC CAC ATA CAC AGC AGC TGT TCA GTA CAA TGT TCT AGA AAA AGA TGA TGA TCC TGC ATC ACT AAC AAT ATG GGT GCC TAT GTT CCA GTC ATC TGT ACC AGC AGA CTT GCT CAT AA	200

[1] Genbank reference sequence accession number.

IAV, Influenza A virus; IBV, Influenza B virus; SC2, SARS-CoV2 virus; RSV, Respiratory Syncytial virus. In the Ultramer™ Duplex Sequence column, italics represent the flanking sequences of the amplicon; single underlines represent the binding sequences of the primers; double underlines represent the binding sequences of the probes.

4. Amplification Efficiency and Multiplex Compatibility

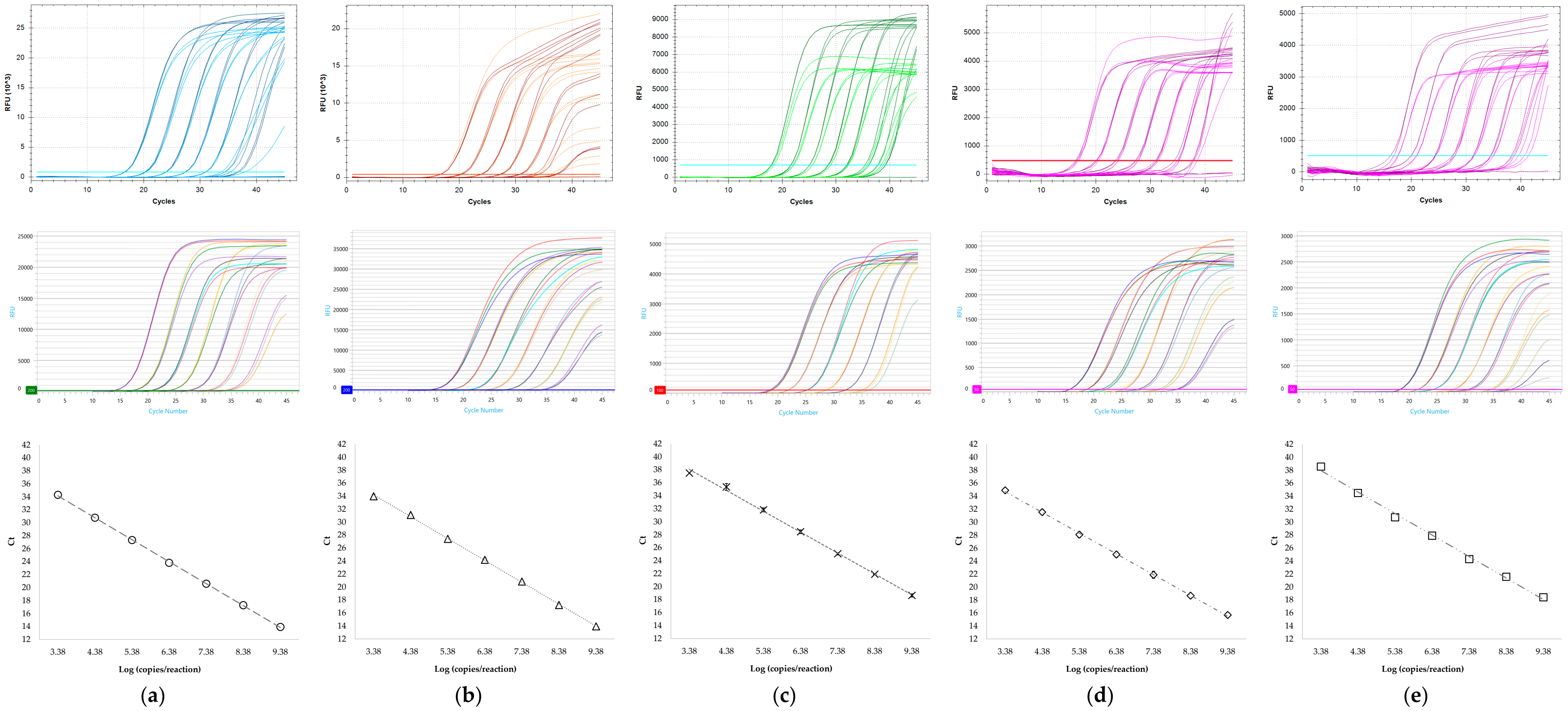

Figure S3. Multiplex compatibility analysis: The top row of the figure displays the fluorescence curves (from CFX96-IVD analyzer) of the multiplex compatibility experiment. In this experiment, light tones represent the multiplex setting, and dark tones represent the monoplex setting of the LDRA assay. The middle row of the figure presents the fluorescence curves (from Panther Fusion® system) of the amplification efficiency verification in a multiplex setting of the LDRA assay. It should be noted that the colors of the curves in this row have no relevant meaning. The bottom row of the figure displays the better linear fit of the Ct values vs. Log(C_i) plot, and the error bars (standard deviation) per level, for amplification efficiency. (a) influenza A virus; (b) influenza B virus; (c) SARS-CoV-2 virus; (d) respiratory syncytial virus, type A; (e) respiratory syncytial virus, type B.

3. In silico *inclusivity*

Coverage was calculated as the relative number of sequences that do not meet the amplification failure prediction criteria (false negatives). To obtain this metric, the relative number of sequences fulfilling at least one of the following criteria was first calculated: i) the number of mismatches (mutations) within the hybridization site exceeds 10% of the oligo length, and ii) there is at least one mismatch within the hybridization site located within the last five nucleotides of the 3′ end of any of the oligos. Inclusivity was calculated as the weighted relative number of sequences that do not meet the amplification failure criteria (Table S3).

Previously published oligo designs homologous to the one discussed in this study are graphically represented in Figure S4.

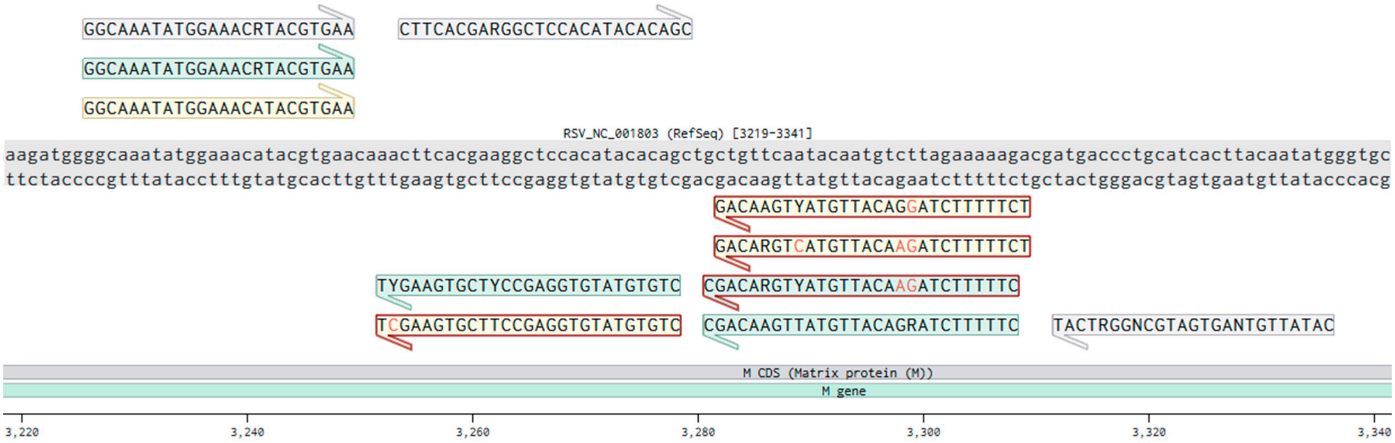

Figure S4. Comparative representation of three sets of oligos for RSV detection. The oligos from this study's design (LDT RSV) are shown in green; the oligos from the design published by Wang, *et al.* [2] * and Tang, *et al.* [3] are shown in yellow; and the oligos from the design published by Fan, *et al.* [4] are shown in gray. The red outline highlights the presence of mismatches, which are specifically located with letters of the same color. The lower axis indicates the region between nucleotides 3,219 and 3,341, taking as reference the Genbank reference sequence with accession number NC_001803.1.

* Of the two yellow reverse primers, only the upper one belongs to the CDC-published design.

For the inclusivity calculation of the RSV A/B oligo designs from this study and those published by Wang, *et al.* [2], Tang, *et al.* [3], and Fan, *et al.* [4], all sequences available up to September 30, 2025 were used: in total, 15,013 RSV A sequences and 16,741–16,747 RSV B sequences. Of these, 3,360 RSV A sequences and 2,834 RSV B sequences were retrieved from the NCBI Virus database (https://www.ncbi.nlm.nih.gov/labs/virus/) and 11,653 RSV A sequences and 13,902–13,907 RSV B sequences from GISAID's EpiRSV™ (https://gisaid.org/). The manufacturers of the comparative Panther Fusion® SARS CoV 2/Flu A/B/RSV assay (Hologic, Inc., CA, USA) [5], reported a total of 1,599 RSV A and 1,240 RSV B sequences between NCBI and GISAID for the period from January 1, 2015 to February 15, 2022 (Figure S5).

Table S3. Inclusivity analysis of the LDT RSV assay.

Sequence set			Assay	Oligo		Sequences with mutations in any region [1]								Sequences with mutations at the 3′ end [1]							Overall Inclusivity (%)
						Thld		> 10 % of mismatches						Thld	≥ 1 mismatch						
Source	Target	N [2]		Type	Size (nt)	Max. Mm (%)	Nt	N / oligo	%	N / target	%	N / assay	%	Max. Mm (nt)	N / oligo	%	N / target	%	N / assay	%	
NCBI [3]	RSV A	3,360	RSV A	RSV A/B_fwd	24	10	2	0	0	0	100	5	99.85	0	0	0	9	99.73	9	99.73	99.73
				RSV A_rev	28		2	0	0						9	0.27					
				RSV A/B_prb	27		2	0	0						0	0					
			RSV B	RSV A/B_fwd	24		2	0	0	5	99.89				0	0	9	99.73			
				RSV B_rev	28		2	5	0.15						9	0.27					
				RSV A/B_prb	27		2	0	0						0	0					
	RSV B	2,834	RSV A	RSV A/B_fwd	24	10	2	0	0	2,145	24.31	2,145	24.31	0	0	0	2	99.93	2	99.93	99.93
				RSV A_rev	28		2	2,145	75.69						2	0.07					
				RSV A/B_prb	27		2	0	0						0	0					
			RSV B	RSV A/B_fwd	24		2	0	0	0	100				0	0	2	99.93			
				RSV B_rev	28		2	0	0						2	0.07					
				RSV A/B_prb	27		2	0	0						0	0					
GISAID [4]	RSV A	11,653	RSV A	RSV A/B_fwd	24	10	2	0	0	0	100	9	99.92	0	2	0.02	109	99.06	109	99.06	99.06
				RSV A_rev	28		2	0	0						107	0.92					
				RSV A/B_prb	27		2	0	0						0	0					
			RSV B	RSV A/B_fwd	24		2	0	0	9	99.92				2	0.02	109	99.06			
				RSV B_rev	28		2	9	0.08						107	0.92					
				RSV A/B_prb	27		2	0	0						0	0					
	RSV B	13,907	RSV A	RSV A/B_fwd	24	10	2	0	0	12,323	11.39	12,323	11.39	0	2	0.01	15	99.89	15	99.89	99.89
				RSV A_rev	28		2	12,323	88.61						13	0.09					
				RSV A/B_prb	27		2	0	0						0	0					
			RSV B	RSV A/B_fwd	24		2	0	0	0	100				2	0.01	15	99.89%			
				RSV B_rev	28		2	0	0						13	0.09					
				RSV A/B_prb	27		2	0	0						0	0					

[1] Criteria for predicting amplification failure (false negatives). [2] Number of sequences after debugging/validation. [3] NCBI Virus database (https://www.ncbi.nlm.nih.gov/labs/virus/). [4] GISAID's EpiRSV™ database (https://gisaid.org/).

N, number of sequences; Nt, nucleotides; Thld, threshold; Max. Mm, maximum mismatches; fwd, forward primer; rev, reverse primer; prb, probe; RSV, respiratory syncytial virus.

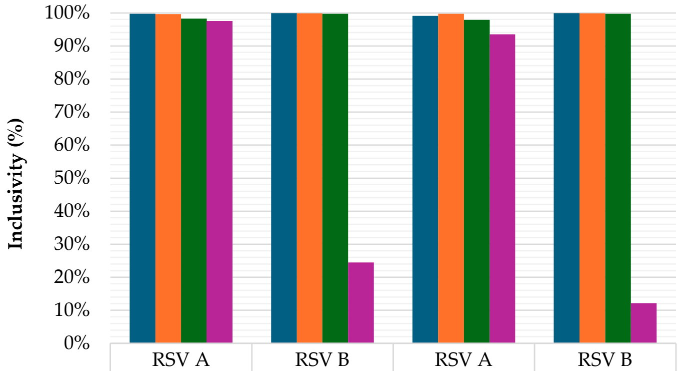

	NCBI		GISAID	
	RSV A	RSV B	RSV A	RSV B
LDT_RSV	99.73%	99.93%	99.06%	99.89%
Fan, et al. 2025	99.64%	99.86%	99.73%	99.86%
Tang, et al. 2024	98.27%	99.75%	97.92%	99.74%
Wang, et al. 2019	97.50%	24.47%	93.50%	12.18%

Database / RSV Type

(**a**)

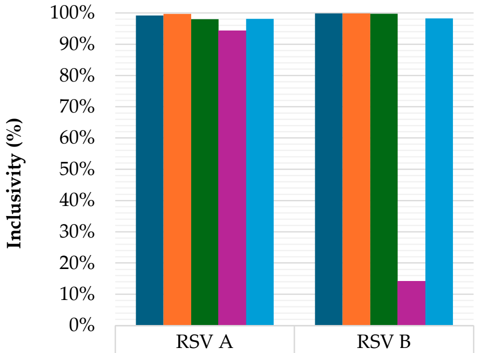

	Pooled	
	RSV A	RSV B
LDT_RSV	99.21%	99.90%
Fan, et al. 2025	99.71%	99.86%
Tang, et al. 2024	98.00%	99.74%
Wang, et al. 2019	94.40%	14.26%
Hologic, 2022	98.12%	98.23%

Database / RSV Type

(**b**)

Figure S5. Comparative inclusivity: (a) Inclusivity by assay, database, and RSV type (A/B); (b) Pooled inclusivity by assay, database, and RSV type (A/B). LDT RSV assay, oligo design for RSV A/B detection of the quadruplex LDRA assay; Fan, *et al.* 2025, oligo design for RSV A/B detection published by [4]; Tang, *et al.* 2024, oligo design for RSV A/B detection published by [3]; Wang, *et al.* 2019, oligo design for RSV A/B detection published by [2]; Hologic, 2022, oligo design for RSV A/B detection of the comparator Panther Fusion® SARS-CoV-2/Flu A/B/RSV assay; NCBI, NCBI Virus database (https://www.ncbi.nlm.nih.gov/labs/virus/); GISAID, GISAID's EpiRSV™ database (https://gisaid.org/); RSV, respiratory syncytial virus types A (RSV A) and B (RSV B). The pooled inclusivity was calculated as the weighted average between databases.

The inclusivity of the primers and probes of the CDC Flu-SC2 assay design is summarized in Table S4.

Table S4. Inclusivity analysis of the CDC Flu-SC2 detection assay.

Sequence set			Oligo		Sequences with mutations in any region [1]						Sequences with mutations at the 3′ end [1]					Overall Inclusivity (%)
					Thld		> 10 % of mismatches				Thld	≥ 1 mismatch				
Source	Target / Assay	N [2]	Type	Size (nt)	Max. Mm (%)	Nt	N / oligo	%	N / target	%	Max. Mm (nt)	N / oligo	%	N / target	%	
GISAID [3]	IAV	177,965	IAV_fwd1/2	25	10	2	0	0	2	99.99	0	453	0.25	486	99.73	99.73
			IAV_rev1/2	23		2	0	0				33	0.02			
			IAV_prb	24		2	2	0.001				0	0			
	IBV	38,339	IBV_fwd	22		2	0	0	0	100	0	422	1.1	428	98.88	98.88
			IBV_rev	21		2	0	0				6	0.02			
			IBV_prb	27		2	0	0				0	0			
	SC2	51,619	SC2_fwd	23		2	0	0	12	99.98	0	43	0.08	104	99.8	99.78
			SC2_rev	25		2	11	0.02				61	0.12			
			SC2_prb *	30		3	1	0.002				0	0			

[1] Criteria for predicting amplification failure (false negatives). [2] Number of sequences after debugging/validation. [3] GISAID's EpiRSV™ database (https://gisaid.org/).

* Modified probe for SC2 detection.

N, number of sequences; Nt, nucleotides; Thld, threshold; Max. Mm, maximum mismatches; fwd, forward primer; rev, reverse primer; prb, probe; IAV, influenza A virus; IBV, influenza B virus; SC2, SARS-CoV-2.

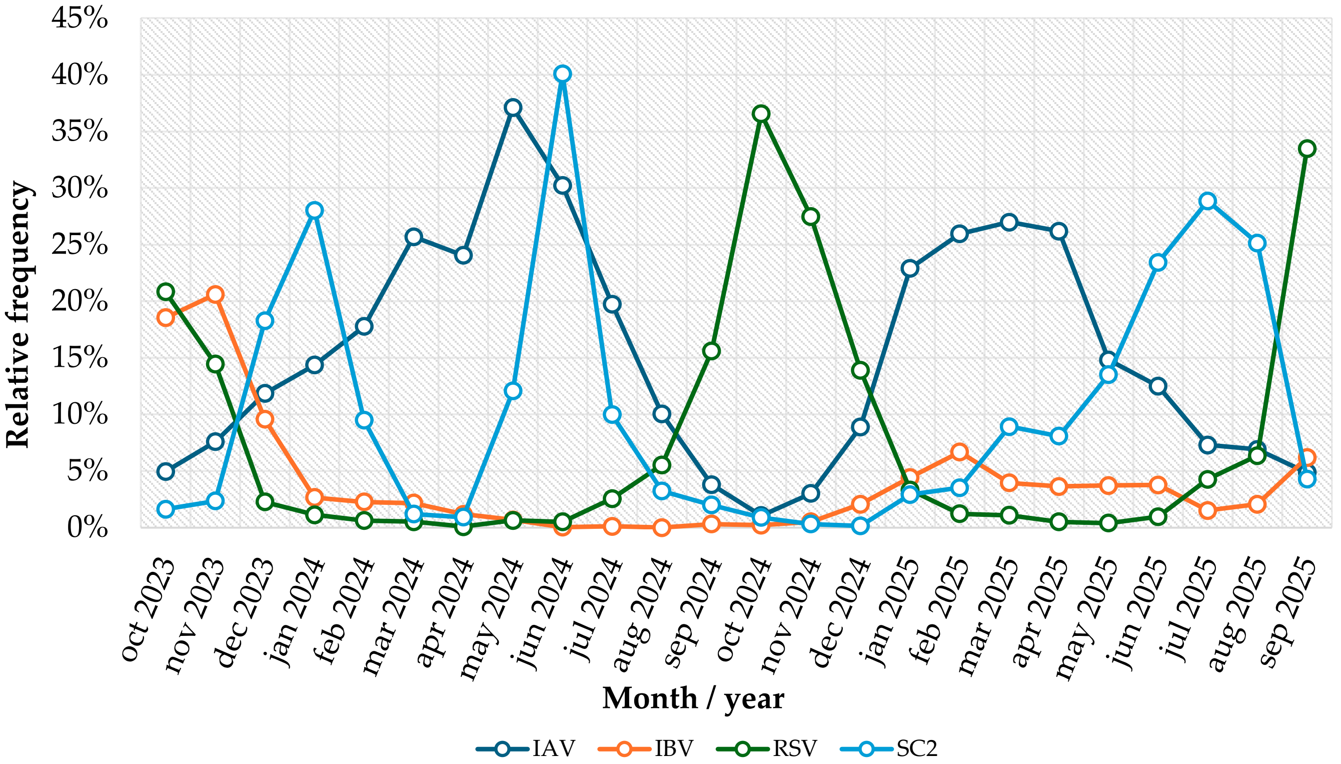

(a)

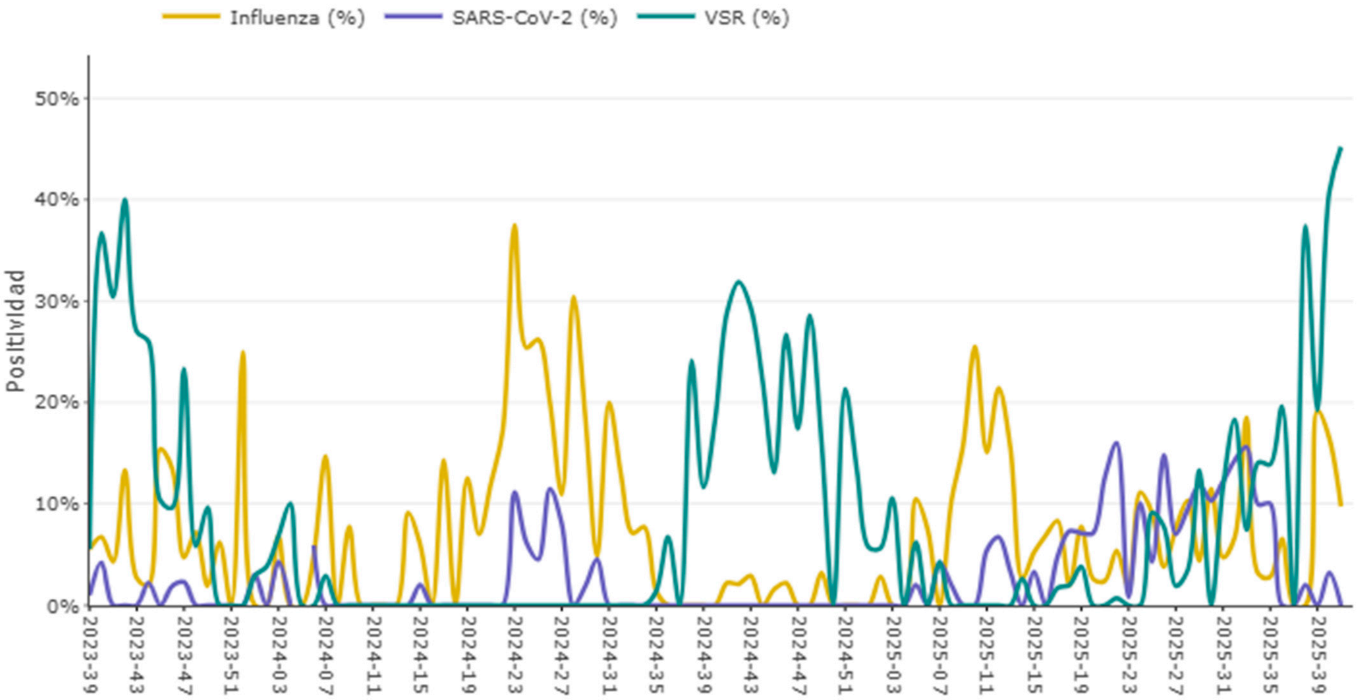

(b)

Figure S6. Positivity (relative frequency) of the influenza A virus (IAV), influenza B virus (IBV), respiratory syncytial virus (RSV) and SARS-CoV-2 (SC2) infections between October 1st, 2023 and September 30th, 2025: (a) data from routine analysis of 26,863 clinical samples from the Molecular Biology Department of the Referencia Laboratorio Clínico, Santo Domingo, Dominican Republic.;

(b) data from the PAHO dashboard for virological surveillance in the Dominican Republic (https://dashboards.pahoflu.com/app/respiratory_viruses/, accessed on September 20th, 2025).

References

1. Caballero Méndez, A.; Reynoso de La Rosa, R.A.; Abreu Bencosme, M.E.; Sosa Ortiz, M.N.; Pichardo Beltré, E.; de La Cruz García, D.M.; Piñero Santana, N.J.; Bacalhau de León, J.C. Screening for Streptococcus agalactiae: Development of an Automated qPCR-Based Laboratory-Developed Test Using Panther Fusion Open AccessTM. *Bio-protocol* **2025**, *15*, e5255, doi:10.21769/BioProtoc.5255.
2. Wang, L.; Piedra, P.A.; Avadhanula, V.; Durigon, E.L.; Machablishvili, A.; López, M.-R.; Thornburg, N.J.; Peret, T.C.T. Duplex real-time RT-PCR assay for detection and subgroup-specific identification of human respiratory syncytial virus. *J. Virol. Methods* **2019**, *271*, 113676, doi:10.1016/j.jviromet.2019.113676.
3. Tang, H.T.; Norz, D.; Grunwald, M.; Giersch, K.; Pfefferle, S.; Fischer, N.; Aepfelbacher, M.; Rohde, H.; Lutgehetmann, M. Analytical and clinical validation of a novel, laboratory-developed, modular multiplex-PCR panel for fully automated high-throughput detection of 16 respiratory viruses. *Journal of clinical virology : the official publication of the Pan American Society for Clinical Virology* **2024**, *173*, 105693, doi:10.1016/j.jcv.2024.105693.
4. Fan, G.; Qian, Q.; Tang, Y.; Liu, J.; Yang, L.; Peng, Y.; Lin, Y.; Ou, G.; Luo, Y.; Shen, C.; et al. The dynamic etiology and epidemiological patterns of acute respiratory tract infections during and post non-pharmacological interventions of SARS-CoV-2 in Shenzhen, China: a two years' prospective cohort study from June 2022. *Frontiers in Cellular and Infection Microbiology* **2025**, *15*, doi:10.3389/fcimb.2025.1599536.
5. Hologic Inc. Panther Fusion® SARS-CoV-2/Flu A/B/RSV Assay. **2025**, *002, AW-32326-001*.
